# Androgen receptor splice variants drive castration-resistant prostate cancer metastasis by activating distinct transcriptional programs

**DOI:** 10.1172/JCI168649

**Published:** 2024-04-30

**Authors:** Dong Han, Maryam Labaf, Yawei Zhao, Jude Owiredu, Songqi Zhang, Krishna Patel, Kavita Venkataramani, Jocelyn S. Steinfeld, Wanting Han, Muqing Li, Mingyu Liu, Zifeng Wang, Anna Besschetnova, Susan Patalano, Michaela J. Mulhearn, Jill A. Macoska, Xin Yuan, Steven P. Balk, Peter S. Nelson, Stephen R. Plymate, Shuai Gao, Kellee R. Siegfried, Ruihua Liu, Mary M. Stangis, Gabrielle Foxa, Piotr J. Czernik, Bart O. Williams, Kourosh Zarringhalam, Xiaohong Li, Changmeng Cai

**Affiliations:** 1 Center for Personalized Cancer Therapy,; 2 Department of Biology, and; 3 Department of Mathematics, University of Massachusetts Boston, Boston, Massachusetts, USA.; 4 Hematology-Oncology Division, Department of Medicine, Beth Israel Deaconess Medical Center and Harvard Medical School, Boston, Massachusetts, USA.; 5 Department of Cell and Cancer Biology, College of Medicine and Life Sciences, The University of Toledo, Toledo, Ohio, USA.; 6 Department of Cell & Developmental Biology, Weill Cornell Medical College, New York, New York, USA.; 7 Human Biology Division, Fred Hutchinson Cancer Center, Seattle, Washington, USA.; 8 Department of Medicine, University of Washington, Seattle, Washington, USA.; 9 Veterans Affairs Puget Sound Health Care System, Geriatric Research and Education Clinical Center (VAPSHCS-GRECC), Seattle, Washington, USA.; 10 Department of Cell Biology and Anatomy and; 11 Department of Biochemistry and Molecular Biology, New York Medical College, Valhalla, New York, USA.; 12 Department of Cell Biology, and Core Technologies and Services, Van Andel Institute, Grand Rapids, Michigan, USA.; 13 Department of Orthopaedic Surgery, MicroCT and Skeletal Research Core Facility, College of Medicine and Life Sciences, The University of Toledo, Toledo, Ohio, USA.

**Keywords:** Oncology, Molecular genetics, Prostate cancer, Transcription

## Abstract

One critical mechanism through which prostate cancer (PCa) adapts to treatments targeting androgen receptor (AR) signaling is the emergence of ligand-binding domain–truncated and constitutively active AR splice variants, particularly AR-V7. While AR-V7 has been intensively studied, its ability to activate distinct biological functions compared with the full-length AR (AR-FL), and its role in regulating the metastatic progression of castration-resistant PCa (CRPC), remain unclear. Our study found that, under castrated conditions, AR-V7 strongly induced osteoblastic bone lesions, a response not observed with AR-FL overexpression. Through combined ChIP-seq, ATAC-seq, and RNA-seq analyses, we demonstrated that AR-V7 uniquely accesses the androgen-responsive elements in compact chromatin regions, activating a distinct transcription program. This program was highly enriched for genes involved in epithelial-mesenchymal transition and metastasis. Notably, we discovered that *SOX9*, a critical metastasis driver gene, was a direct target and downstream effector of AR-V7. Its protein expression was dramatically upregulated in AR-V7–induced bone lesions. Moreover, we found that Ser81 phosphorylation enhanced AR-V7’s pro-metastasis function by selectively altering its specific transcription program. Blocking this phosphorylation with CDK9 inhibitors impaired the AR-V7–mediated metastasis program. Overall, our study has provided molecular insights into the role of AR splice variants in driving the metastatic progression of CRPC.

## Abstract 2

<em>AR</em> splice variants promote osteoblastic bone lesions of prostate cancer by transcriptionally activating a specific metastasis program that is distinct from the full-length AR activity.

## Graphical abstract

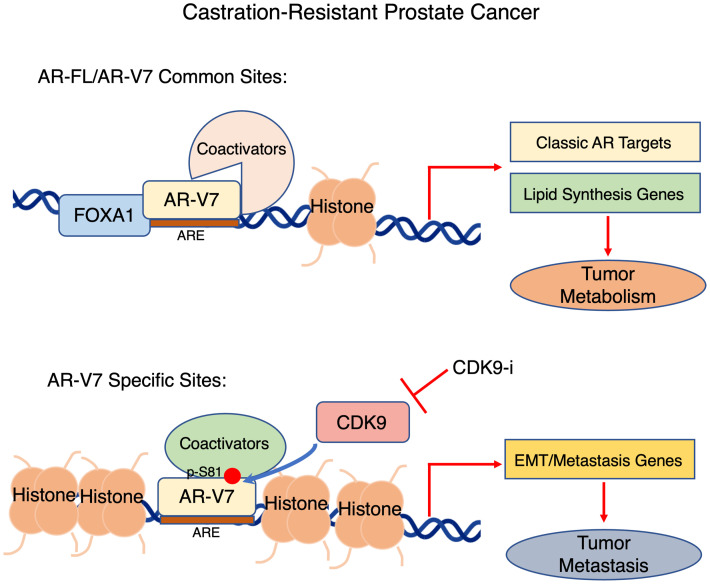

## Introduction

The androgen receptor (AR) is critical in driving prostate cancer (PCa) development, with androgen deprivation therapy (ADT) being the standard treatment for PCa patients. Although initial responses are generally positive, cancers often relapse, progressing to the castration-resistant stage of PCa (CRPC) with partially restored AR signaling (1, 2). Even with more aggressive AR signaling inhibition treatments (ARSi), such as enzalutamide or abiraterone, most patients eventually develop resistance (3, 4). In those resistant tumors, a subset progresses through AR-independent mechanisms, but the majority relapse via regaining AR activity, facilitated by various mechanisms, including AR gene alterations, amplifications, mutations, aberrant splicing, and dysregulation of cofactors (5). A key mechanism involves increased expression of ligand-binding domain–truncated (LBD-truncated) and constitutively active AR splice variants (AR-Vs), predominantly AR-V7 (6–9) and less frequently ARv567es (10, 11). These variants enable ligand-independent activities, regulating gene transcription in CRPC cells. AR-Vs have been shown to activate AR signaling without androgens, thus enabling PCa cells to adapt to therapies targeting full-length AR (AR-FL) (12–14). However, the role of AR-Vs in regulating in vivo development of more aggressive PCa forms, such as metastasis, by activating transcription programs distinct from AR-FL, is not well understood.

Numerous studies have highlighted the role of AR-Vs in driving CRPC resistance to ARSi (10, 11, 15–21). After ADT and ARSi treatments, there is a rapid and significant increase in AR-V7 expression. This increase is attributed to the disruption of the negative feedback loop that regulates AR gene expression as well as further changes in its splicing (8, 9, 21, 22). Notably, prostate-specific overexpression of AR-V7 or ARv567es in transgenic mouse models induces prostatic intraepithelial neoplasia or invasive carcinoma by activating oncogenic transcription programs (11, 23). This is in sharp contrast with early findings where AR-FL overexpression did not induce prostate neoplasia (24), suggesting distinct biological functions for AR-Vs. In fact, increased AR-V expression in CRPC versus castration-sensitive PCa (CSPC) bone metastases samples correlates with poor prognosis (25). Initial mechanistic studies suggested that AR-V7 primarily heterodimerizes with AR-FL to enhance AR-FL activity in low-androgen conditions (19, 26). However, recent studies clearly show that AR-V7 can independently regulate its transcription targets (14, 17–19). The debate continues as to whether AR-Vs maintain distinct transcription programs by binding to non–AR-FL-occupied chromatin sites (13, 16–20, 26). For instance, using the CWR-22Rv1 model (expressing very high levels of AR-V7), one study found that AR-V7 drives a unique program by binding to distinct sites, interacting with the transcription factor ZFX (17). Another study using a CRPC cell line (LNCaP-95) derived from lymph node carcinoma of the prostate (LNCaP) expressing endogenous AR-V7 showed significant overlap in chromatin binding between AR-V7 and AR-FL, and further demonstrated that the lack of LBD allows AR-V7 to preferentially interact with corepressors, potentially differing in function from AR-FL (20). Yet, these studies did not identify distinct biological functions of AR-V7 compared with AR-FL.

To more precisely compare the activity of AR-V7 with that of AR-FL, we generated lentiviral stable CRPC cell lines with inducible overexpression of either AR-V7 or AR-FL. We then assessed the differential effects on tumor cell metastasis by injecting these cells into zebrafish embryos and the tibias of castrated male mice. Our data show that overexpressing AR-V7, but not AR-FL, in CRPC cells promotes tumor cell invasion into blood vessels and induces osteoblastic bone lesions in vivo. Integrated analyses of ChIP-seq, ATAC-seq, and RNA-seq revealed a previously undefined chromatin activity of AR-V7. This activity enables AR-V7 to bind to androgen-responsive elements (AREs) within compact chromatin, thereby activating a unique transcription program. This program is independent of AR-FL activity and highly enriched for genes mediating epithelial-mesenchymal transition (EMT) and metastasis functions. The analysis also identified a subset of unique AR-V7 targets, including the stem cell and metastatic factor *SOX9*, which we previously reported as playing a role in promoting PCa progression and metastasis (27, 28). Furthermore, we demonstrated that the Ser81 phosphorylation of AR-V7 selectively enhances its unique pro-metastasis function. Targeting this phosphorylation with CDK9 inhibitors can block AR-V7–induced *SOX9* expression and its associated metastasis function.

## Results

### Overexpression of AR-V7, but not AR-FL, induces osteoblastic bone lesions in PCa.

**Figure 1:**
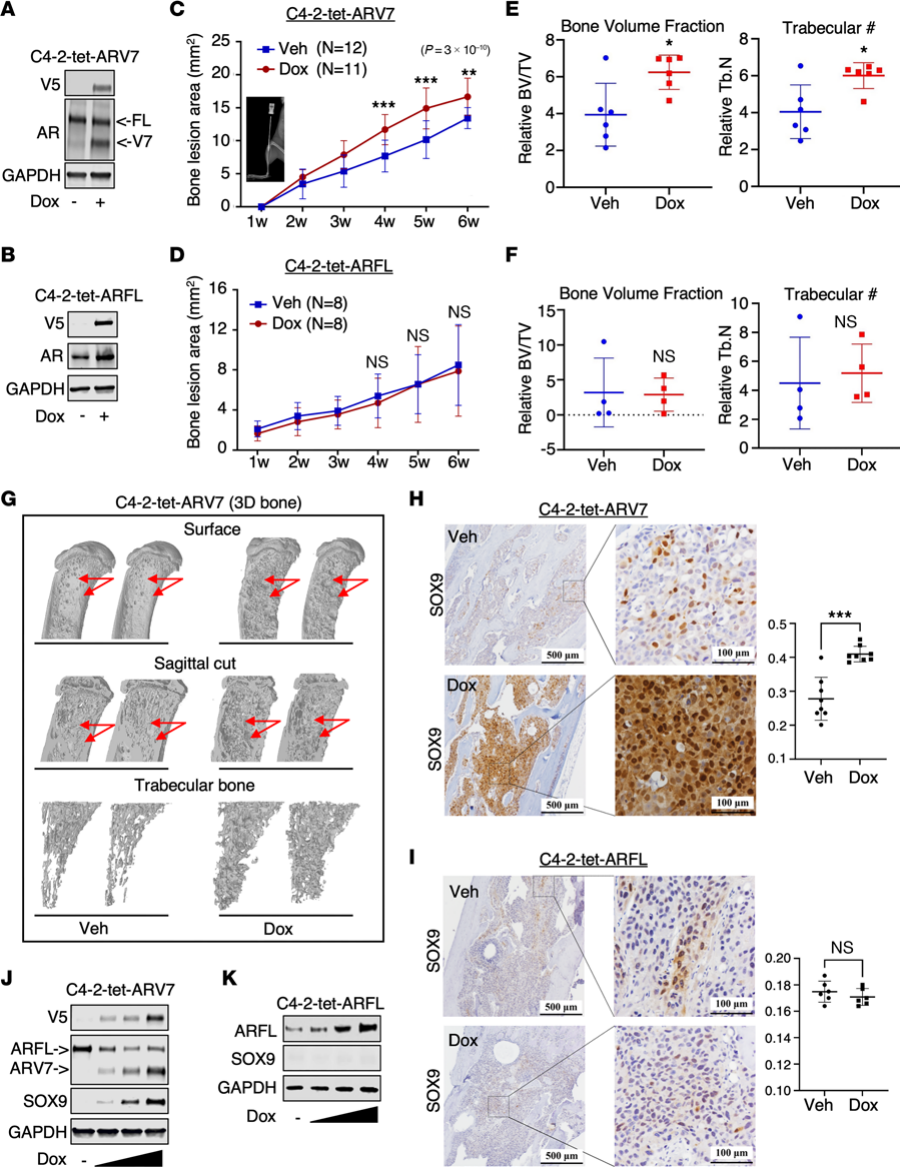
Overexpression of AR-V7, but not AR-FL, induces osteoblastic bone metastasis in PCa. (**A** and **B**) Immunoblotting for AR-V7 or AR-FL in C4-2–derived lentiviral stable lines overexpressing doxycycline-regulated, V5-tagged AR-V7 (C4-2-tet-ARV7) (**A**) or AR-FL (C4-2-tet-ARFL) (**B**). Cells were pretreated with or without 0.25 μg/mL doxycycline for 48 hours. (**C** and **D**) C4-2-tet-ARV7 (**C**) or C4-2-tet-ARFL (**D**) cells were injected into the tibias of castrated NSG mice, which were then fed with or without a doxycycline-supplemented diet. The bone lesion area was monitored and quantified. (**E** and **F**) Normalized bone volume (BV) and trabecular bone number (Tb.N) in C4-2-tet-ARV7 tumors (**E**) or C4-2-tet-ARFL tumors (**F**) were compared. TV, total volume. (**G**) Structural views of bones scanned by μCT and 3D reconstructed for the C4-2-tet-ARV7 model. (**H** and **I**) Immunohistochemistry (IHC) staining for SOX9 in tumor samples from the C4-2-tet-ARV7 model (**H**) and the C4-2-tet-ARFL model (**I**). Scale bars (**H** and **I**): 500 μm (left) and 100 μm (right). (**J** and **K**) Immunoblotting for AR (antibody against N-terminus) and SOX9 in C4-2-tet-ARV7 (**J**) and C4-2-tet-ARFL cells (**K**), which were treated with 0, 0.1, 0.5, or 1 μg/mL doxycycline for 48 hours. All the cell lines were hormone depleted prior to the experiments. Unpaired, 2-sided Student’s *t* test was used to determine statistical significance. *P < 0.05, **P < 0.01, ***P < 0.001.

PCa frequently metastasizes to bone. To directly compare the functions of AR-V7 and AR-FL in bone metastasis, we generated 2 lentiviral stable lines expressing doxycycline-regulated V5-tagged AR-V7 (C4-2-tet-ARV7) or AR-FL (C4-2-tet-ARFL) using the C4-2 PCa cell line, which was derived from castration-resistant LNCaP xenografts and does not express AR-V7 (Figure 1, A and B). The induced expression levels of AR-V7 in C4-2-tet-ARV7 cells were similar to the endogenous levels of AR-FL in these cells and comparable to the overexpressed levels of AR-FL in C4-2-tet-ARFL cells. We next assessed the effect of AR-V7 on bone metastasis by injecting these cells into the tibias of castrated male mice. As shown in Figure 1, C–F, and Supplemental Figure 1, A and B (supplemental material available online with this article; https://doi.org/10.1172/JCI168649DS1), the expression of AR-V7, but not AR-FL, markedly increased the area of bone lesions at 4–6 weeks after injection, and induced osteoblastic bone formation characterized by a significant increase in bone volume fraction and trabecular bone number. Micro-CT and 3D reconstruction revealed massive bone destruction, typical of mixed osteoblastic and osteolytic bone lesions, on the surface and inside of the tibias injected with AR-V7–inducing cells, but not in uninduced cells or cells overexpressing AR-FL (Figure 1G and Supplemental Figure 1C). We also examined SOX9 expression, a well-studied metastasis driver (29–32), in these bone lesions. As shown in Figure 1, H and I, SOX9 expression dramatically increased in tumors expressing AR-V7, but remained low and unchanged in tumors overexpressing AR-FL. Consistently, SOX9 protein expression appeared to be induced by AR-V7 expression, but not AR-FL, in cell culture (Figure 1, J and K). A similar effect on bone metastasis was also observed in the AR-V–positive CWR-22Rv1 model with the use of an RNAi approach to target AR-V7 (Supplemental Figure 1D). These findings suggest a potential role for AR-V7 in promoting osteoblastic bone metastasis, specifically in the stages of bone colonization and outgrowth, highlighting its significance in CRPC progression.

### AR-V7 activates a unique transcription program enriched for EMT and metastasis functions.

**Figure 2:**
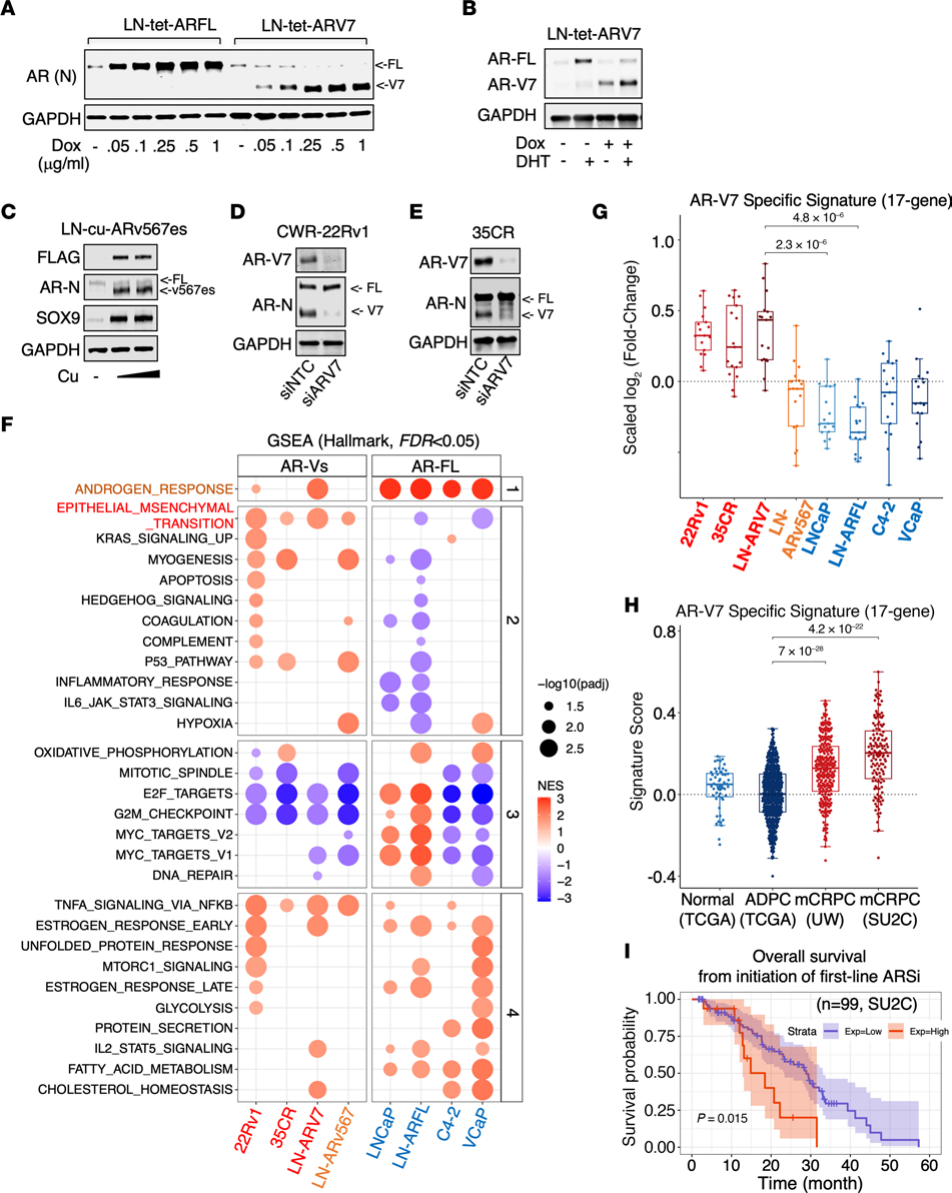
AR-V7 activates a unique transcription program in CRPC. (**A**) Immunoblotting for AR (N-terminus) in LNCaP cells stably expressing doxycycline-regulated V5-tagged AR-FL (LN-tet-ARFL) or AR-V7 (LN-tet-ARV7) treated with 0–1 μg/mL doxycycline for 48 hours. (**B**) Immunoblotting for AR in LN-tet-ARV7 cells treated with or without low-dose doxycycline (0.1 μg/mL for 48 hours) or DHT (10 nM for 24 hours). (**C**) Immunoblotting for indicated proteins in LNCaP cells stably expressing cumate-regulated FLAG-tagged ARv567es (LN-cu-ARv567es) with the treatment of 0, 30, or 60 μg/mL cumate for 48 hours. (**D** and **E**) Immunoblotting for AR-V7 and N-terminal AR in CWR-22Rv1 (**D**) and LuCaP 35CR cells (**E**) transfected with siRNAs against nontarget control (NTC) or AR-V7 for 3 days. (**F**) RNA-seq analyses were conducted to compare the AR-V7 transcriptome (22Rv1 and 35CR transfected with siNTC or siARV7 for 3 days, LN-tet-ARV7 treated with or without 0.25 μg/mL doxycycline for 48 hours) with the ARv567es transcriptome (LN-cu-ARv567es treated with or without 30 μg/mL cumate for 48 hours) and DHT-stimulated AR-FL transcriptome (LNCaP/C4-2/VCaP stimulated with or without 10 nM DHT for 24 hours, LN-tet-ARFL treated with 0.25 μg/mL doxycycline and stimulated with or without 0.1 nM DHT for 24 hours). GSEA normalized enrichment scores (NES) of MSigDB Hallmark gene sets in each model were plotted (red, AR-V7– or AR-FL–activated pathways; blue, AR-V7– or AR-FL–repressed pathways). All the cell lines were hormone depleted prior to the experiments. (**G** and **H**) The expression of AR-V7–specific targets (17-gene signature) in these cell lines (**G**) and in human PCa cohorts: Normal (*n* = 52) and androgen-dependent primary PCa samples (*n* = 498) from TCGA data set versus metastatic CRPC samples from SU2C (*n* = 266) and UW data sets (*n* = 138) (**H**). *P* values are shown above the horizontal bars. (**I**) Kaplan-Meier survival analysis for the overall survival from the initiation of the first-line ARSi in mCRPC patients (SU2C cohort, *n* = 99) was conducted, comparing top 25th percentile of median score expression (red, *n* = 25) versus lower 75th percentile (blue, *n* = 74). *P* value was calculated using the log-rank test from the score test. Statistical analyses were conducted using unpaired, nonparametric 2-sample Wilcoxon’s test for box-and-whisker plots, with Bonferroni’s correction for multiple comparisons.

Given our functional studies, we hypothesized that AR-V7 regulates a distinct transcription program, activating the metastatic cascade in bone. Since AR-FL expression is consistently upregulated upon castration and its overexpression alone in CSPC cells is capable of inducing resistance to castration (33, 34), we then generated lentiviral stable lines expressing doxycycline-regulated V5-tagged AR-V7 (LN-tet-ARV7) and AR-FL (LN-tet-ARFL) using the androgen-sensitive LNCaP cell line. Low-dose doxycycline treatment induced AR-V7 to levels comparable to androgen-stimulated endogenous AR-FL in LN-tet-ARV7 cells, but lower than overexpressed AR-FL in LN-tet-ARFL cells (Figure 2, A and B, and Supplemental Figure 2, A and B). As a comparison to AR-V7, we also generated an LNCaP-derived stable cell line that expresses cumate-regulated FLAG-tagged ARv567es (LN-cu-ARv567es), which upon cumate treatment, induced SOX9 protein expression similarly (Figure 2C). The options for CRPC cell line models to study endogenous AR-V7 are limited, with CWR-22Rv1 being the primary model. To address this issue, we established a new AR-V7–expressing CRPC cell line by adapting a LuCaP 35CR patient-derived xenograft (PDX) (35) to tissue culture. The established 35CR cell line exhibits high levels of AR-V7, which can be dramatically reduced by high-level androgen treatment, aligning with our previous findings (9) (Supplemental Figure 2C).

RNA-seq analyses were conducted on LN-tet-ARV7 cells treated with or without doxycycline to identify the AR-V7 transcriptome. Similarly, analyses were performed on LN-tet-ARFL cells treated with doxycycline and stimulated with or without 0.1 nM dihydrotestosterone (DHT, to mimic castration levels of DHT) to identify the AR-FL transcriptome under castrated conditions. The results were compared with the AR-V7 transcriptome in 22Rv1 and 35CR cells (with AR-V7 silencing) (Figure 2, D and E), the ARv567es transcriptome in LN-cu-ARv567es cells (treated with cumate), the AR-FL transcriptome in parental LNCaP cells (stimulated with 10 nM DHT), and our previously published AR-FL transcriptome data in C4-2 and VCaP CRPC cell lines (stimulated with 10 nM DHT) (36, 37). Gene set enrichment analyses (GSEA) using hallmark gene sets revealed that while AR-V7– and AR-FL–activated genes were similarly enriched for the classic androgen response pathway in most models, genes activated by AR-V7 or ARv567es in all the models were specifically enriched for the EMT functions, a pathway not activated by AR-FL in any of these PCa models (Figure 2F). Interestingly, AR-V7 also appears to exert transcriptional repression function, similar to AR-FL stimulated by high-level androgens, targeting many shared targets and enriching for E2F signaling and cell cycle pathways (38, 39) (Supplemental Figure 3, A–C). This repressive activity aligns with findings from a prior study (40), though it varies from others (41), likely due to differing experimental conditions or models. To further explore the unique activity of AR-V7 in patient samples, we developed a 17-gene signature representing AR-V7 specifically activated genes (Figure 2G and Supplemental Figure 4A). The scores of this signature were significantly increased in metastatic CRPC (mCRPC) patient cohorts compared with primary hormone–dependent PCa cohorts (42–45) (Figure 2H and Supplemental Figure 4B). Furthermore, patients within the top quartile scores of this signature experienced significantly worse clinical outcomes compared with the others (Figure 2I), suggesting that these distinct AR-V7 targets may play crucial roles in CRPC progression.

**Figure 3:**
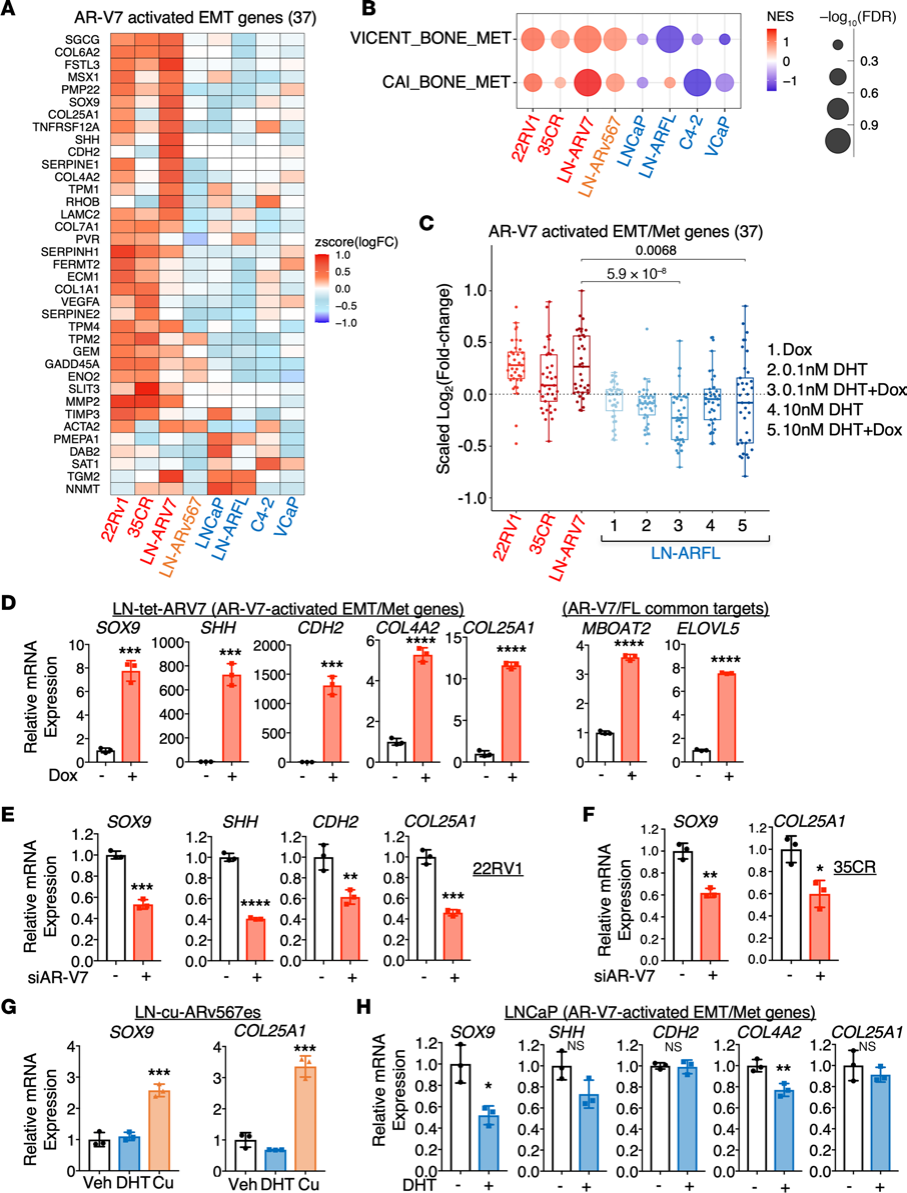
AR-V7–activated transcription program is enriched for EMT/metastasis functions. (**A**) Heatmap view of identified AR-V7–activated EMT genes (37-gene signature). (**B**) GSEA NES of 2 public bone metastasis signatures (VICENT_BONE_MET, 20-gene; CAI_BONE_MET, 44-gene) in each model were plotted (red, activated pathways; blue, repressed pathways). Statistical analyses were conducted using unpaired, nonparametric 2-sample Wilcoxon’s test for box-and-whisker plots, with Bonferroni’s correction for multiple comparisons. (**C**) Fold-change of AR-V7–activated EMT genes (37-gene signature) in 22RV1, 35CR, LN-tet-ARV7, and LN-tet-ARFL cells. *P* values by Wilcoxon’s test are shown above the horizontal bars. (**D**) qRT-PCR for a panel of AR-V7–regulated genes in LN-tet-ARV7 cells treated with or without 0.25 μg/mL doxycycline. (**E** and **F**) qRT-PCR for a panel of AR-V7–regulated genes in 22Rv1 (**E**) or 35CR (**F**) cells transfected with siNTC or siARV7. (**G**) qRT-PCR for *SOX9* and *COL25A1* in LN-cu-ARv567es cells treated with 10 nM DHT for 24 hours or 30 μg/mL cumate for 48 hours. (**H**) qRT-PCR for a panel of AR-V7–regulated genes in parental LNCaP cells treated with or without 10 nM DHT for 24 hours. All the cell lines were hormone depleted prior to the experiments. For the bar graphs, an unpaired, 2-sided Student’s *t* test was used to determine statistical significance. *P < 0.05, **P < 0.01, ***P < 0.001, ****P < 0.0001. Data are represented as mean ± SEM.

Next, we identified a panel of 37 genes specifically activated by AR-V7 in EMT pathways, including *SOX9* and *SHH*, the latter of which is also known to promote cancer metastasis (46, 47) (Figure 3A). Intriguingly, ARv567es activates only approximately 25% of these EMT genes. Considering the role of many EMT genes in metastasis, we conducted further GSEA using 2 bone metastasis signatures derived from lung cancer and PCa (48, 49). This analysis revealed strong enrichments in the transcriptomes of AR-V7 and ARv567es, but not consistently in AR-FL (Figure 3B). We then examined the androgen regulation of this gene subset using our recently published RNA-seq analyses in LN-tet-ARFL cells treated with varying combinations of doxycycline and DHT (low versus high) (50). As shown in Figure 3C, under none of these conditions did AR-FL broadly activate the expression of this metastasis-associated gene subset, although a small fraction might be upregulated by high-dose androgen treatment (10 nM DHT) in the context of AR overexpression.

Using LN-tet-ARV7, 22Rv1, and 35CR cells, we confirmed the regulation of *SOX9* and several other identified metastasis genes by AR-V7, along with 2 previously identified common targets in lipid biosynthesis pathways (*MBOAT2*, *ELOVL5*) shared by AR-FL and AR-V7 (51) (Figure 3, D–F). This upregulation is not a nonspecific response to doxycycline treatment, as shown in Supplemental Figure 5A. Additionally, we validated the upregulation of the *SOX9* gene by ARv567es (Figure 3G). However, the expression of those metastasis targets was not increased by androgen-stimulated AR-FL, whereas the expression of lipid synthesis targets was androgen-induced (Figure 3H and Supplemental Figure 5B). Overall, these results suggest that LBD-truncated AR variants, such as AR-V7, distinctly activate specific transcription programs, which are strongly enriched for EMT and metastasis functions.

### AR-V7 can bind to a subset of chromatin sites distinct from AR-FL binding.

**Figure 4:**
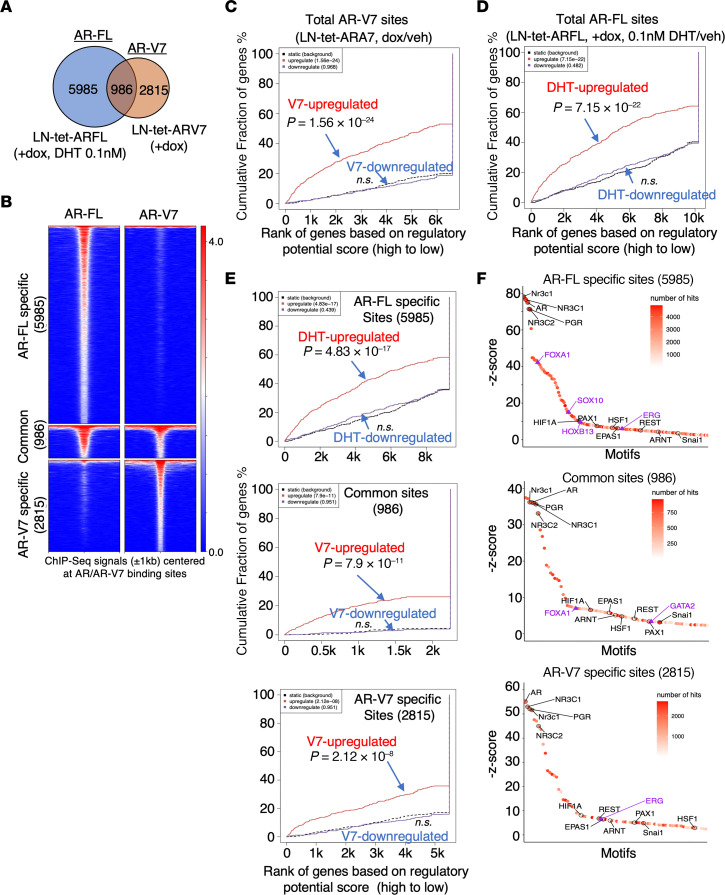
AR-V7 can bind to a subset of chromatin sites distinct from AR-FL binding. (**A** and **B**) ChIP-seq analysis of V5 was conducted in LN-tet-ARV7 cells (hormone depleted) stimulated with or without 0.25 μg/mL doxycycline for 48 hours. Similarly, ChIP-seq analysis of AR (antibody against N-terminus) was performed in LN-tet-ARFL cells stimulated with 0.25 μg/mL doxycycline and then treated with 0.1 nM DHT for 4 hours. The Venn diagram (**A**) and heatmap view (**B**) demonstrate the unique or overlapping sites of AR-V7 versus AR-FL. (**C**–**E**) Binding and expression target analysis (BETA) was used to assess the association of total AR-V7 sites with AR-V7–regulated genes (**C**), total AR-FL sites with androgen-upregulated genes (**D**), and the unique or common sites of AR-FL/AR-V7 with androgen-upregulated genes or AR-V7–regulated genes (**E**). *P* values were calculated by BETA as a measure of the significance of the association between transcription factor binding and gene expression changes. (**F**) Motif enrichment analyses were conducted for the AR-FL/AR-V7 unique or common sites and ranked by *z* score (black, common enriched motifs; purple, uniquely enriched motifs).

To elucidate the molecular basis for the distinct AR-V7 activity, we performed V5 ChIP-seq in LN-tet-ARV7 cells under hormone-depleted conditions to examine AR-V7 chromatin binding and identified 3,801 high-confidence binding peaks (Supplemental Figure 6A). We next performed ChIP-seq on total AR-FL in LN-tet-ARFL cells treated with doxycycline and stimulated by low-dose DHT to determine the AR-FL cistrome under castrated conditions and identified 6,971 high-confidence peaks. We then compared the AR-V7 cistrome with AR-FL and found 986 overlapping sites (Figure 4, A and B). Next, we conducted a binding and expression target analysis (BETA) (52) to examine the association of AR-V7/AR-FL binding with their regulated genes. As shown in Figure 4, C and D, AR-V7 and AR-FL total chromatin binding sites were all highly associated with their transcription activation function. Importantly, AR-V7/AR-FL common binding sites and AR-V7 unique sites were all strongly associated with AR-V7–activated genes (*P* = 7.9 × 10^–11^, *P* = 2.12 × 10^–8^, respectively) (Figure 4E), indicating AR-V7 binding at its specific sites are also transcriptionally active. We next examined enriched motifs at the AR-V7–specific sites versus the common or AR-FL–specific sites. Consistent with previous findings (17–19), AR binding motifs were top-ranked in all AR-V7 sites as well as AR-FL sites (Figure 4F). The motif of FOXA1, a critical pioneer factor for AR access to chromatin (53), was highly enriched in AR-FL–specific and the common sites, but not in AR-V7–specific binding sites. This finding is consistent with previous reports (17, 18) and implies that the chromatin structure at AR-V7–specific sites may be different.

Several studies have proposed that the major activity of AR-V7 in CRPC is to promote AR-FL chromatin binding under castrated conditions through heterodimerization with AR-FL (13, 16, 26). Therefore, we next determined whether inducing AR-V7 expression can increase endogenous AR-FL binding by performing ChIP-seq analyses on AR-FL in LN-tet-ARV7 cells. As shown in Supplemental Figure 6B, AR-V7 induction did not significantly change the chromatin binding of AR-FL in the absence of DHT treatment, but markedly decreased the number of AR-FL binding sites in the presence of DHT. We then identified 403 and 1,100 AR-FL/AR-V7 co-occupied sites in the absence or presence of DHT, respectively (Supplemental Figure 6C). The intensity of basal AR-FL binding was not significantly changed in either AR-FL unique sites or AR-FL/V7 co-occupied sites (Supplemental Figure 6D), indicating that AR-V7 cannot increase the chromatin recruitment of AR-FL in the absence of androgens. Interestingly, while the binding intensity of DHT-stimulated AR-FL at the co-occupied sites was not significantly changed by AR-V7, a strong repressive effect on AR-FL binding at AR-FL unique sites was observed (Supplemental Figure 6D), suggesting that there was a possible indirect repressive effect on AR-FL binding by AR-V7. The repressed AR-FL binding also led to markedly reduced chromatin accessibility (Supplemental Figure 6E). We noticed that the protein expression of AR-FL was repressed by AR-V7, particularly in the presence of androgen treatment (Figure 2B). Since our early studies showed that AR-FL can function as a transcriptional repressor to repress its own gene expression through binding to a suppressive site at its intron 2 and recruiting a repressor complex (36), it is likely that AR-V7 may maintain this repressor function and can transcriptionally repress AR-FL. This model was supported by further experiments showing that the mRNA level of endogenous AR-FL was indeed repressed by AR-V7 (Supplemental Figure 6F). Nonetheless, these findings suggest that AR-V7 may not enhance the chromatin binding of AR-FL in CRPC cells.

### AR-V7 can bind to compact chromatin regions.

**Figure 5:**
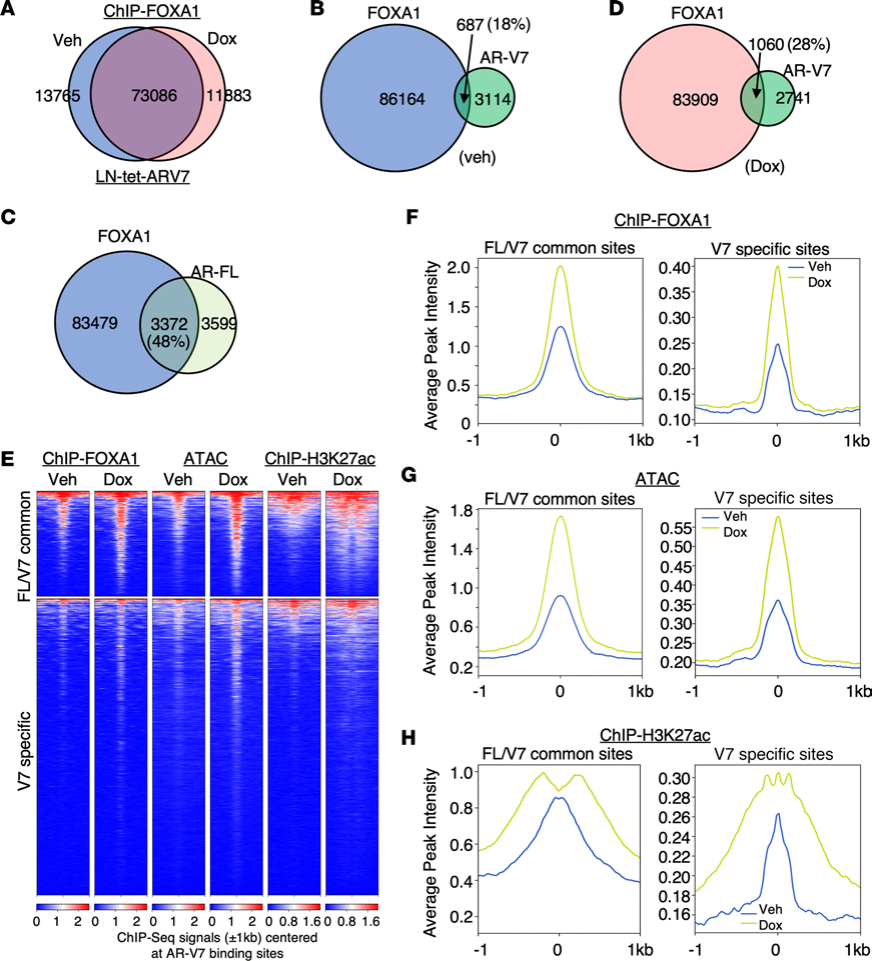
AR-V7 can bind to more compact chromatin regions. (**A**–**D**) ChIP-seq analyses of FOXA1 were conducted in LN-tet-AR-V7 cells treated with or without 0.25 μg/mL doxycycline for 48 hours. Venn diagrams demonstrate FOXA1 binding sites in treated versus untreated cells (**A**), FOXA1 binding sites in untreated cells versus AR-V7 sites (**B**), FOXA1 binding sites in untreated parental LNCaP cells versus AR-FL binding sites (**C**), and FOXA1 binding sites in doxycycline-treated cells versus AR-V7 sites (**D**). (**E**) ATAC-seq and ChIP-seq analyses of H3K27ac were performed in LN-tet-AR-V7 cells treated with or without 0.25 μg/mL doxycycline for 48 hours. The heatmap shows peak intensity of FOXA1, ATAC, and H3K27ac at AR-FL/AR-V7 common sites or AR-V7–specific sites. (**F**–**H**) The average intensity curves of FOXA1 (**F**), ATAC (**G**), and H3K27ac (**H**) at AR-FL/AR-V7 common sites versus AR-V7–specific sites. All the cell lines were hormone depleted prior to the experiments.

FOXA1, a pioneer transcription factor, functions to decompact chromatin structure through its winged-helix forkhead DNA binding domain, thereby facilitating the binding of AR and the estrogen receptor (54, 55). To examine the levels of FOXA1 binding at AR-V7 unique sites, we performed ChIP-seq of FOXA1 in LN-tet-ARV7 cells treated with or without doxycycline. The results showed that AR-V7 overexpression did not globally alter the FOXA1 chromatin binding (Figure 5A). Consistent with motif enrichment analysis, only approximately 18% of AR-V7 binding sites were pre-occupied by FOXA1 compared with approximately 48% for AR-FL binding sites (Figure 5, B and C). Interestingly, AR-V7 binding was found to increase FOXA1 occupancy at AR-V7 binding sites from approximately 18% to approximately 28% (Figure 5D). Notably, the average FOXA1 binding intensity at AR-V7–specific sites was much lower than at common sites prior to AR-V7 chromatin binding (Figure 5, E and F). This suggests that AR-V7 is capable of binding to cryptic AREs that are less enriched for putative FOXA1 binding motifs and typically inaccessible to AR-FL, and that its binding at these sites can subsequently stimulate FOXA1 binding.

To further investigate chromatin structure at AR-V7 binding sites, we performed ATAC-seq to examine chromatin accessibility and ChIP-seq of acetylated histone 3 lysine 27 (H3K27ac) to evaluate enhancer activation in LN-tet-ARV7 cells. The results showed that the average intensity signals of ATAC and H3K27ac were approximately 2- to 3-fold lower at AR-V7–specific sites compared with common sites. However, these signals notably increased upon AR-V7 binding (Figure 5, E, G, and H). These findings were corroborated at specific AR-V7 binding sites near the 37 EMT/metastasis genes and validated at several target gene sites (Supplemental Figure 7, A–D). Collectively, these data indicate that AR-V7 uniquely binds to AREs within compact chromatin, subsequently promoting FOXA1 binding, enhancing chromatin accessibility, and activating enhancers.

### AR-V7 transcriptionally activates the SOX9 gene.

**Figure 6:**
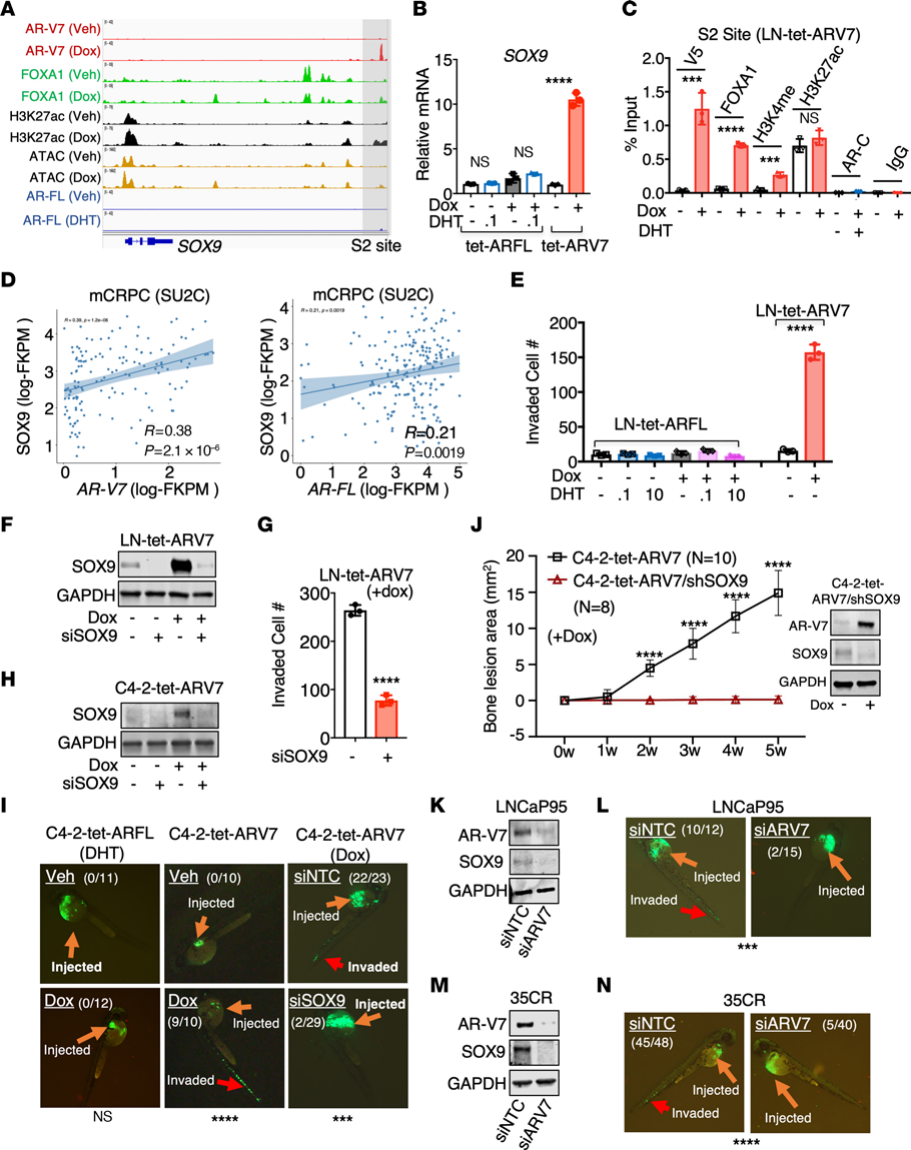
AR-V7 transcriptionally activates *SOX9*. (**A**) Genome browser view for indicated protein binding at the S2 site of the *SOX9* gene (Note: AR-FL binding indicates AR ChIP-seq peaks in LN-tet-ARFL cells treated with doxycycline; other tracks are for LN-tet-ARV7 cells). (**B**) qRT-PCR for *SOX9* mRNA in LN-tet-ARFL cells (0.1 nM DHT for 24 hours, 0.25 μg/mL doxycycline for 48 hours) and in LN-tet-ARV7 (0.25 μg/mL doxycycline for 48 hours). (**C**) ChIP-qPCR for V5 (AR-V7), FOXA1, H3K4me2, H3K27ac, and C-terminal AR (AR-FL) at the S2 site in LN-tet-ARV7 cells treated with/out 0.25 μg/mL doxycycline for 48 hours or 0.1 nM DHT for 4 hours. (**D**) Spearman’s correlation of *SOX9* expression with *AR-V7* or *AR-FL* expression in the SU2C mCRPC data set. (**E**) Matrigel invasion assay in LN-tet-ARV7 cells (doxycycline) compared with LN-tet-ARFL cells (0–10 nM DHT, 0.1 μg/mL doxycycline for 3 days). (**F** and **G**) Immunoblotting for SOX9 (**F**) and Matrigel invasion assay (**G**) in LN-tet-ARV7 cells transfected with siNTC or siSOX9 for 3 days. (**H**) Immunoblotting for SOX9 in GFP-labeled C4-2-tet-ARV7 cells transfected with siNTC or siSOX9 for 3 days. (**I**) GFP-labeled C4-2-tet-ARFL (grown under 0.1 nM DHT) or C4-2-tet-ARV7 stable cells, pretreated with or with out 0.25 μg/mL doxycycline and transfected with siNTC or siSOX9 for 3 days, were injected into the zebrafish embryos. AR-V7–mediated tumor cell intravasation process was observed within 1 hour (indicated by red arrow). The proportion of invaded embryos relative to the total number of embryos injected is displayed. (**J**) C4-2 cells stably expressing doxycycline-regulated AR-V7 together with doxycycline-regulated shRNA against SOX9 (LN-tet-ARV7/shSOX9) were established. Immunoblotting for AR-V7 and SOX9 was performed (right panel). LN-tet-ARV7/shSOX9 or control LN-tet-ARV7 cells were then injected into the tibias of castrated male mice, which were then fed with a doxycycline-supplemented diet. The bone lesion area was monitored and quantified (left panel). (**K** and **L**) Immunoblotting for AR-V7 and SOX9 (**K**) and zebrafish embryo metastasis assay (**L**) in GFP-labeled LNCaP-95 cells transfected with siNTC or siARV7 for 3 days. (**M** and **N**) Immunoblotting for AR-V7 and SOX9 (**M**) and zebrafish embryo metastasis assay (**N**) in GFP-labeled 35CR cells transfected with siNTC or siARV7 for 3 days. All the cell lines were hormone depleted prior to the experiments. ***P < 0.001; ****P < 0.0001 by unpaired, 2-sided Student’s *t* test (**B**, **C**, **E**, **G**, and **J**), Fisher’s exact test (**K** and **L**), or χ^2^ test (**N**). Data are represented as mean ± SD.

Among the identified AR-V7 targets, the stem cell and metastasis transcription factor SOX9 plays a critical role in PCa development (27). In developing prostate, SOX9 is expressed by epithelial cells invading into urogenital sinus mesenchyme, and its loss results in profound defects in prostate ductal morphogenesis in mouse (56). In *Pten*-deficient mouse prostate, we and other groups have demonstrated that prostate-specific *Sox9* overexpression promotes the development of invasive carcinoma (27, 57). Examination of published data sets (18, 20, 43, 44, 58, 59) revealed consistently increased *SOX9* expression in metastatic CRPC compared with primary PCa and its upregulation by AR-V7 (Supplemental Figure 8, A–C). Previously, we identified a cryptic ARE site in the 3′ downstream region of the *SOX9* gene, termed the S2 site (Figure 6A), which, in cooperation with nearby transcription factors such as ERG, regulates *SOX9* expression under high-dose androgen stimulation (27). CRISPR-mediated transcriptional activation (CRISPRa) of this site significantly increased *SOX9* expression (Supplemental Figure 8D). Using quantitative real-time reverse transcription PCR (qRT-PCR), we confirmed that *SOX9* expression is strongly activated by AR-V7 but not by castration-level androgen-stimulated AR-FL (Figure 6B and Supplemental Figure 8, E and F). AR-V7 strongly binds to the FOXA1-low S2 site, inducing FOXA1 recruitment and increasing the levels of H3K4me2 and possibly H3K27ac (Figure 6C). In the SU2C mCRPC data set, *SOX9* expression was more strongly associated with *AR-V7* than *AR-FL* (Figure 6D).

Interestingly, while DHT-stimulated AR-FL can increase LNCaP cell proliferation, AR-V7 did not stimulate cell growth (Supplemental Figure 9, A and B), suggesting that AR-V7 specifically promotes metastasis rather than proliferation. To further investigate whether SOX9 is a downstream effector of AR-V7’s pro-metastasis function, we performed in vitro Matrigel invasion assays. The results showed that AR-FL only slightly increased cell invasion, whereas AR-V7 expression dramatically promoted it (Figure 6E and Supplemental Figure 9C). Notably, this AR-V7–induced cell invasion was markedly repressed by SOX9 silencing (Figure 6, F and G, and Supplemental Figure 9D), suggesting a critical role of SOX9 in CRPC invasion and metastasis.

In a zebrafish metastasis model, we further assessed whether SOX9 mediates the pro-metastasis function of AR-V7 in vivo. Zebrafish do not develop an adaptive immune system until 14 days after fertilization, and can be used to rapidly evaluate cancer cell intravasation and dissemination, early steps in the metastasis cascade (60). The GFP-labeled AR-V7– and AR-FL–overexpressing C4-2 cells were injected into zebrafish embryos (~10–20 per group) to examine their metastatic potential. AR-V7–expressing cells invaded the blood vessel within an hour after injection (9/10 versus 0/10 invaded embryos), whereas cells overexpressing AR-FL under DHT stimulation did not invade and stayed within the perivitelline space of each embryo (0/12 versus 0/11 invaded embryos) (Figure 6, H and I). Notably, silencing SOX9 in AR-V7–expressing cells dramatically prevented invasion (2/29 versus 22/23 invaded embryos). Moreover, overexpressing SOX9 alone was sufficient to drive LNCaP cell metastasis (8/10 versus 0/10 invaded embryos) (Supplemental Figure 9, E and F), highlighting the critical pro-metastasis function of SOX9 in PCa. Finally, we tested the involvement of SOX9 in AR-V7–induced bone lesion formation using the C4-2-tet-ARV7 cell line, now stably incorporating a doxycycline-inducible shRNA targeting SOX9 (C4-2-tet-ARV7/shSOX9). In this model, doxycycline treatment induces AR-V7, but simultaneously prevents SOX9 induction (Figure 6J). Remarkably, silencing SOX9 in this context dramatically inhibited the formation of bone lesions induced by AR-V7 (Figure 6J). In fact, this alteration completely halted bone lesion development, indicting a pivotal role of SOX9 in mediating PCa tumor metastasis driven by AR-V7.

Additionally, we tested whether endogenous AR-V7 expression similarly promotes metastasis. Unlike AR-V7–negative parental LNCaP cells or LNCaP-derived C4-2 cells, LNCaP-95 cells, expressing high levels of AR-V7 (20), exhibited strong invasive and metastatic capabilities. Silencing AR-V7 markedly reduced the metastatic activity of these cells both in vitro and in vivo (Figure 6, K and L, and Supplemental Figure 9G). A similar effect was also observed in the 35CR model (Figure 6, M and N). Overall, these data clearly indicate that SOX9 is a critical downstream effector of AR-V7 in promoting CRPC metastasis.

### Ser81 phosphorylation is required for AR-V7–induced metastasis.

**Figure 7:**
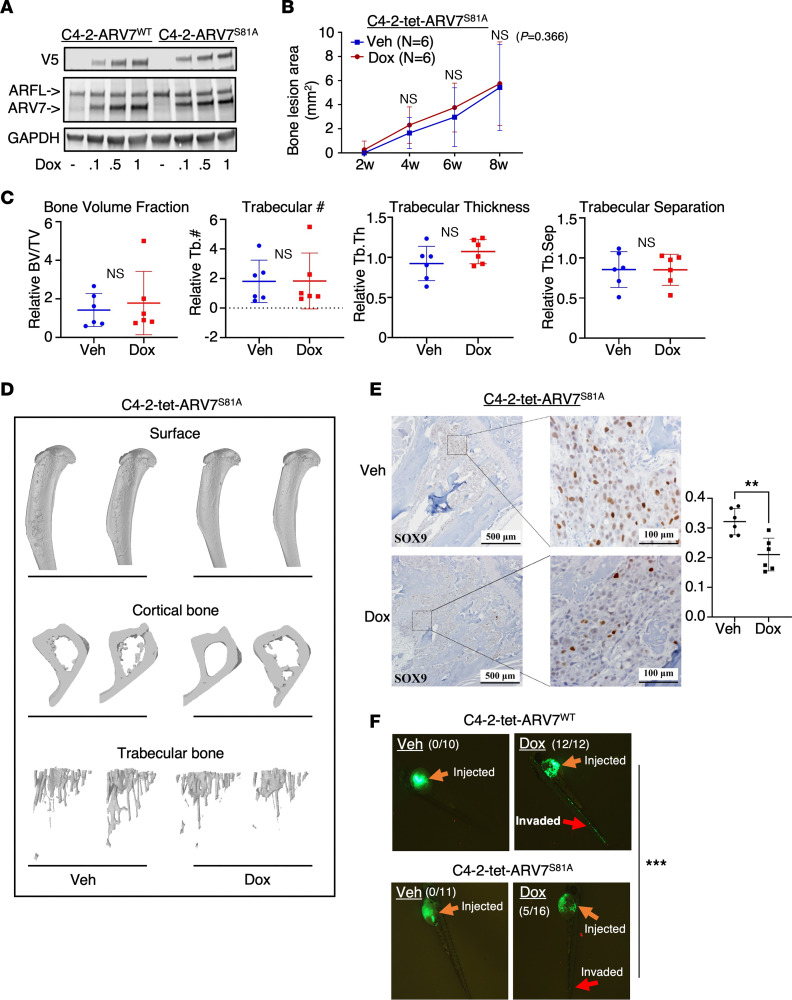
Ser81 phosphorylation is required for AR-V7–induced metastasis. (**A**) Immunoblotting for indicated proteins in C4-2-tet-ARV7 and C4-2-ARV7^S81A^ cells (C4-2 cells expressing doxycycline-regulated V5-tagged AR-V7-S81A mutant) treated with 0–1 μg/mL doxycycline for 48 hours. (**B**) C4-2-ARV7^S81A^ cells were injected into the tibias of castrated male mice, which were then fed with or without a doxycycline-supplemented diet. The bone lesion area was monitored and quantified. Note: this experiment was conducted simultaneously with the C4-2-tet-ARV7 and C4-2-tet-ARFL experiments shown in Figure 1. (**C**) Normalized bone volume and trabecular bone number were compared. (**D**) Structure views of bones scanned by μCT and 3D reconstructed. (**E**) IHC staining for SOX9 in tumor samples. Scale bars: 500 μm (left) and 100 μm (right). (**F**) Zebrafish embryo metastasis assay in GFP-labeled C4-2-tet-ARV7^WT^ and C4-2-tet-ARV7^S81A^ cells pretreated with or without 0.25 μg/mL doxycycline for 48 hours. All the cell lines were hormone depleted before experiments. **P < 0.01; ***P < 0.001 by unpaired, 2-sided Student’s *t* test (**B**, **C**, and **E**) or Fisher’s exact test (**F**).

We next examined whether posttranslational modifications of the AR-V7 protein may affect its activity. A candidate for such modification is Ser81 phosphorylation, previously reported to increase AR-FL protein stability, chromatin binding, and transcription activity (61–64). A recent study also suggests that this modification is associated with AR reactivation in CRPC (65). However, the role of Ser81 phosphorylation in regulating AR-V7 function remains unclear. To investigate, we created a loss-of-function S81A mutant in C4-2 cells. Intriguingly, this mutation led to slower migration of the AR-V7 protein, suggesting that a second posttranslational modification might be tightly linked with S81 dephosphorylation (Figure 7A). We then injected these cells into the tibias of castrated mice to assess the impact of the S81A mutation on bone metastasis. Remarkably, the S81A mutant failed to induce osteoblastic bone lesions, showing no increase in bone lesion area, bone volume fraction, trabecular bone numbers, or bone surface/cavity destruction (Figure 7, B–D), suggesting a defect in its pro-metastasis function. We also examined SOX9 protein expression in the bone lesions and observed no induction of SOX9 expression (Figure 7E), in sharp contrast to the wild-type (WT) AR-V7 activity (Figure 1). Furthermore, we conducted a zebrafish embryo metastasis assay comparing cells expressing WT AR-V7 and the S81A mutant. As shown in Figure 7F, the mutant displayed substantially impaired metastatic activity compared with the WT (5/16 versus 12/12 invaded embryos). These findings clearly demonstrate the critical role of S81 phosphorylation in maintaining the pro-metastasis function of AR-V7.

### Ser81 phosphorylation selectively enhances the AR-V7–regulated metastasis program.

**Figure 8:**
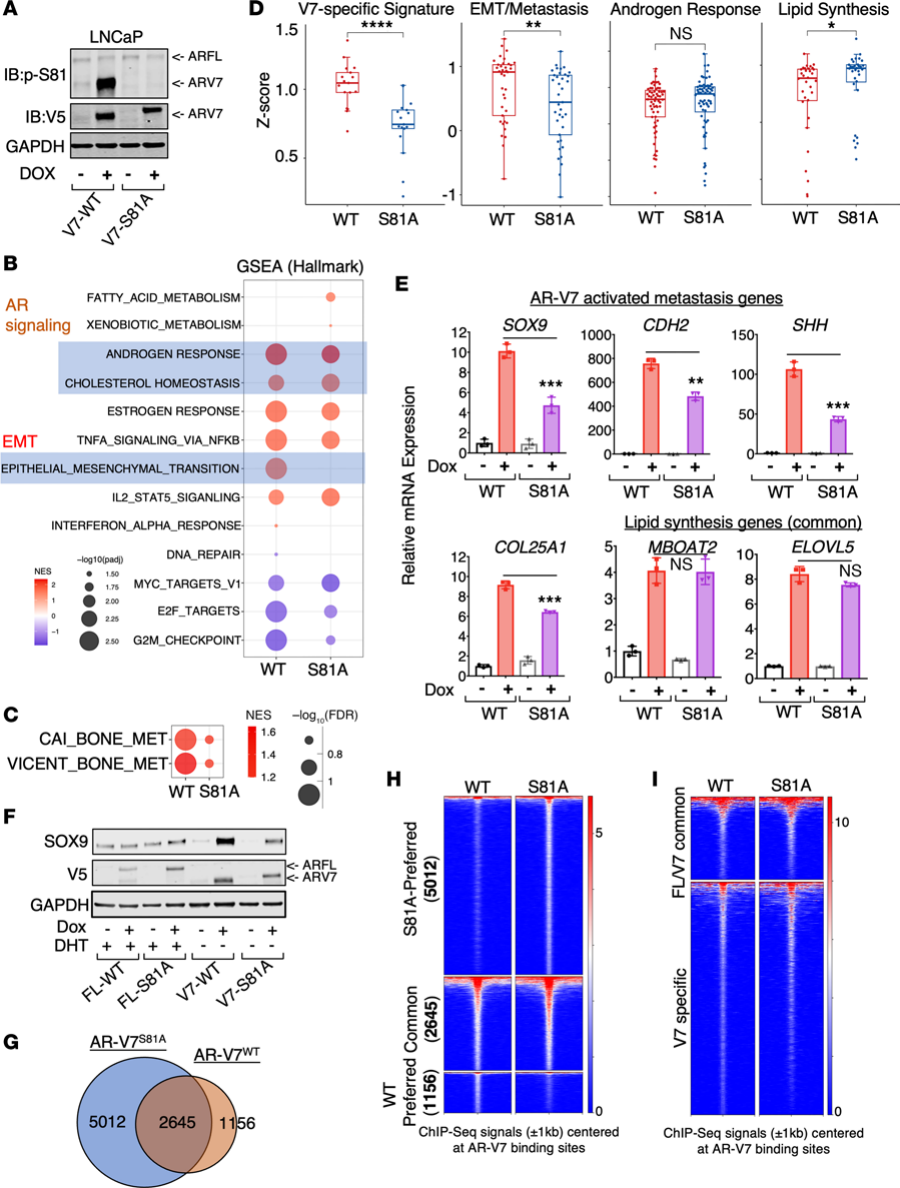
Ser81 phosphorylation selectively enhances the AR-V7–regulated metastasis program. (**A**) Immunoblotting for S81-phosphorylated (p-S81) AR-FL and AR-V7 in LN-tet-ARV7 and LN-tet-ARV7^S81A^ cells (LNCaP cells expressing doxycycline-regulated V5-tagged AR-V7-S81A mutant). (**B** and **C**) RNA-seq analyses were conducted in these stable lines treated with or without 0.25 μg/mL doxycycline. GSEA using Hallmark gene sets (**B**) or predefined bone metastasis gene sets (**C**) was performed. (**D**) Relative fold change for AR-V7 regulation of indicated gene sets. (**E**) qRT-PCR for AR-V7–activated EMT/metastasis genes and lipid synthesis genes. (**F**) Immunoblotting for SOX9 and AR in LN-tet-ARFL, LN-tet-ARFL^S81A^, LN-tet-ARV7, and LN-tet-ARV7^S81A^ cells, treated with 10 nM DHT or 0.25 μg/mL doxycycline. (**G**–**I**) ChIP-seq analysis of V5 was performed in LN-tet-ARV7^S81A^ cells stimulated with or without 0.25 μg/mL doxycycline. The Venn diagram for AR-V7-WT bindings sites versus AR-V7-S81A binding sites (**G**), heatmap view for peak intensity at AR-V7-WT and AR-V7-S81A unique or common sites (**H**), and heatmap view for peak intensity at AR-V7 and AR-FL unique or common sites (**I**) are shown. All the cell lines were hormone depleted before experiments. *P < 0.05; **P < 0.01; ***P < 0.001; ****P < 0.0001 by Wilcoxon’s test (**D**) or unpaired, 2-sided Student’s *t* test (**E**). Data are represented as mean ± SD.

We then created an S81A mutation in LN-tet-ARV7 cells to study its molecular function in regulating AR-V7. As shown in Figure 8A, S81 was highly phosphorylated in WT AR-V7 protein but not endogenous AR-FL under hormone-depleted conditions. The slower migration of AR-V7-S81A proteins was consistently observed in this model, and it was not affected by the addition of phosphatase (Supplemental Figure 10A). Interestingly, overexpressing the AR-FL-S81A mutant in LNCaP cells did not indicate any significant alteration in AR-FL protein movement during electrophoresis (Supplemental Figure 10B), suggesting that this effect is specific to AR-V7 protein. Nonetheless, we performed RNA-seq analyses to determine the effect of the S81A mutation on the AR-V7 transcriptome. As shown in Figure 8, B and C, while the enrichment of the classic AR signaling pathway was barely affected, the enrichment of EMT and bone metastasis functions were markedly repressed by the S81A mutant. This suggests that S81 phosphorylation may selectively enhance this distinct transcription program of AR-V7. Indeed, the overall expression levels of AR-V7 specifically activated genes (17-gene signature) or AR-V7–regulated EMT/metastasis genes (37-gene signature) were significantly decreased by the S81A mutation, while the levels of classic androgen-regulated genes and previously identified AR-FL/AR-V7–regulated lipid biosynthesis pathway genes were not suppressed (Figure 8D and Supplemental Figure 10, C and D). Consistently, AR-V7–induced expression of SOX9 and other metastasis genes was significantly decreased by the S81A mutant, while AR-V7–induced lipid synthesis genes were not affected (Figure 8, E and F).

To further determine whether the altered AR-V7 transcription program by the S81A mutant is due to decreased chromatin binding at its specific sites, we performed ChIP-seq analyses of AR-V7-WT and AR-V7-S81A. Surprisingly, decreased AR-V7 binding by the S81A mutant was only observed at a small fraction of binding sites, and the overall AR-V7 binding intensity at the AR-FL/AR-V7 common binding sites or AR-V7 specific sites was not notably changed (Figure 8, G–I). Interestingly, we also observed a large amount of gained binding sites (5,012 sites) associated with AR-V7-S81A. The genes annotated with these sites were functionally enriched for adherens junction and Hippo signaling pathways (Supplemental Figure 10, E and F). Consistent with these global findings, the AR-V7 binding and its associated FOXA1 binding at the SOX9-S2 site were not significantly changed (Supplemental Figure 10, G and H). These data suggest that S81 phosphorylation may affect AR-V7 activity through mechanisms independent of chromatin binding.

### CDK9 inhibition prevents phosphorylation of AR-V7 Ser81 and impairs AR-V7–mediated metastasis.

**Figure 9:**
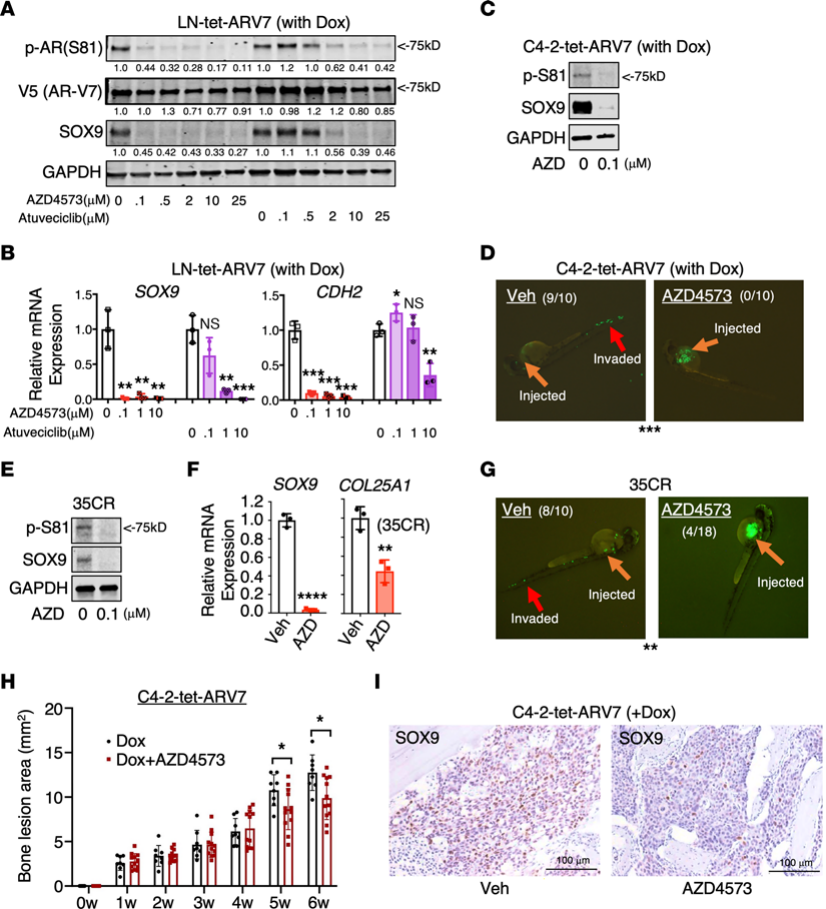
CDK9 inhibition prevents Ser81 phosphorylation of AR-V7 and impairs AR-V7–mediated metastasis. (**A**) Immunoblotting for p-S81 of AR-V7, V5, and SOX9 in LN-tet-ARV7 cells treated with CDK9 inhibitors for 24 hours. (**B**) qRT-PCR for AR-V7 target genes in LN-tet-ARV7 cells treated with 2 CDK9 inhibitors for 24 hours. (**C** and **E**) Immunoblotting for p-S81 of AR-V7 and SOX9 in C4-2-tet-ARV7 (**C**) and 35CR (**E**) cells treated with or without 0.1 μM AZD4573 for 24 hours. (**D** and **G**) Zebrafish embryo metastasis assays in GFP-labeled C4-2-tet-ARV7 cells cultured under doxycycline (**D**) and 35CR cells (**G**) pretreated with or without 0.1 μM AZD4573 for 24 hours. (**F**) qRT-PCR for AR-V7 target genes in 35CR cells treated with 0.1 μM AZD4573 for 24 hours. (**H**) C4-2-tet-ARV7 cells were injected into the tibias of castrated male mice, which were then fed with a doxycycline-supplemented diet and treated with (*n* = 12) or without AZD4573 (*n* = 8, 15 mg/kg) via i.p. injection every other day. The bone lesion area was monitored and quantified. (**I**) IHC staining for SOX9 in tumor samples. Scale bars: 100 μm. All the cell lines were hormone depleted before experiments. *P < 0.05; **P < 0.01; ***P < 0.001; ****P < 0.0001 by unpaired, 2-sided Student’s *t* test (bar graphs) or χ^2^ test (**D** and **G**). Data are represented as mean ± SD.

Prior studies have identified S81 on AR-FL as a phosphorylation site for CDK1 and CDK9 (61, 62). However, we found that while CDK1 inhibitors effectively blocked the S81 phosphorylation in AR-FL, they do not have the same effect on AR-V7 (Supplemental Figure 11A). This observation led us to explore the potential role of CDK9 in phosphorylating S81 on AR-V7 and to examine whether inhibiting CDK9 could suppress the metastasis-promoting activities of AR-V7. Our experiments with LN-tet-ARV7 cells, treated with 2 clinically tested CDK9 inhibitors, AZD4573 and atuveciclib (66, 67), revealed that these inhibitors abolished S81 phosphorylation on the AR-V7 protein and markedly reduced the expression of key AR-V7 target genes, such as *SOX9* and *CDH2* (Figure 9, A and B). Notably, while higher doses of AZD4573 might affect AR-V7 expression levels, lower doses had minimal effect on its protein and mRNA expression (Figure 9A and Supplemental Figure 11B). Furthermore, treatment with low-dose AZD4573 nearly completely inhibited the metastatic capability of C4-2-tet-ARV7 cells in the zebrafish model (Figure 9, C and D). A similar effect was also observed in the LuCaP 35CR model (Figure 9, E–G). To assess the efficacy of inhibiting CDK9 in the context of AR-V7–induced bone lesions, we treated mice bearing intratibial C4-2-tet-ARV7 tumors using AZD4573. The results, as demonstrated in Figure 9, H and I, showed that AZD4573 treatment reduced the formation of bone lesions induced by AR-V7 and markedly suppressed SOX9 expression. Collectively, these findings support the further exploration of CDK9 inhibitor treatment as a strategy to target the AR-V7–induced metastasis cascade in CRPC.

## Discussion

It is now relatively clear that the elevated expression of AR-V7, and possibly other AR-Vs, is a major driving force for the partially restored AR signaling in CRPC adapted to ADT or ARSi treatments. However, the question of whether AR-Vs can drive a distinct transcription program favoring CRPC tumor progression, due to their structural differences from AR-FL, remains debated. In this study, we hypothesized that AR-V7 not only functions as a mimic of AR-FL in sustaining AR signaling in response to ARSi, but also plays a critical role in activating a distinct transcription program to further promote more aggressive PCa progression. To test this hypothesis, we generated stable PCa lines expressing doxycycline-inducible AR-V7 or AR-FL to mimic the increased AR-FL/AR-V7 expression in CRPC and injected these cells into the tibias of castrated male mice. Notably, we found that AR-V7 can induce severe osteoblastic bone lesions under castrated conditions, while overexpressed AR-FL fails to do so. This functional difference between AR-FL and AR-V7 was also observed in vitro using a Matrigel invasion assay and in vivo using a zebrafish embryo metastasis assay, indicating that AR-Vs may have a unique activity in accelerating the metastasis cascade in PCa. Using a combined analysis of ChIP-seq and RNA-seq, we then identified a distinct AR-V7 transcription program that is highly enriched for genes involved in EMT and metastasis functions, findings consistent with early transgenic mouse studies (11, 23).

Importantly, we identified *SOX9*, a critical stem cell and metastasis driver gene, as a direct target of AR-Vs, and its transcription is tightly regulated by AR-V7–mediated activation of a previously identified cryptic ARE site (S2 site) (27). The protein expression of SOX9 was also dramatically upregulated in AR-V7–induced bone lesions, but not in AR-FL–expressing tumors. While AR-V7 can also activate additional metastasis regulators, such as *SHH*, the AR-V7/SOX9 axis appears to be a critical signaling event for inducing metastasis, as silencing SOX9 markedly reduced the metastatic capability of AR-V7–expressing PCa cells. Given the important function of *SOX9* in regulating cancer stem cells (29), it is plausible that initial ADT/ARSi treatment induces AR-V7 expression, which subsequently activates *SOX9* to maintain metastatic cancer stem cells, possessing both stem cell properties and invasive capabilities that contribute to cancer metastasis. Interestingly, our early studies on *SOX9* have indicated a *TMPRSS2-ERG*–mediated AR reprogramming that can also activate *SOX9* expression through binding to the same cryptic ARE site under high-androgen conditions (27). Therefore, SOX9 may be initially activated by *TMPRSS2-ERG* and high-dose androgen–stimulated AR-FL in androgen-dependent PCa cells. After ADT treatments, androgen levels and ERG expression dramatically decrease, and thus the PCa cells are unable to sustain SOX9 expression and metastasis. However, the acute increase in AR-V expression may quickly take control of the regulation of *SOX9*, more broadly and robustly activating *SOX9* even in fusion-negative PCa tumors. Future studies can determine whether ERG may also be involved in enhancing AR-V7–mediated *SOX9* activation.

We also want to emphasize a recent comprehensive study on pan-metastatic cancers, including mCRPC, that identified 2 distinct metastasis subtypes: proliferative and EMT-like (68). Proliferative metastatic tumors show increased proliferation, metabolism, and stress response, whereas EMT-like metastatic tumors are marked by EMT and inflammation-related signatures. This suggests that mCRPC tumors, to adapt to the bone environment, might display a reduced cell proliferation signature along with an enhanced EMT signature, consistent with our findings on the metastatic driver role of AR-V7. Intriguingly, AR-FL might drive the proliferative metastasis through its upregulation of cell cycle and metabolic processes. Nonetheless, further research is needed to thoroughly understand the interplay between proliferation and EMT pathways in bone metastasis.

Consistent with other studies, our ChIP-seq analyses identified common chromatin binding sites accessible to both AR-V7 and liganded AR-FL. These sites are enriched for the FOXA1 binding motif and located in open chromatin structures. Examining the endogenous AR-FL binding in LN-tet-ARV7 cells, we found that AR-V7 and AR-FL may co-occupy a subset of chromatin sites. However, our data did not indicate any major enhancement effect of AR-V7 in facilitating AR-FL chromatin binding. Thus, whether AR-V7 forms a heterodimer with AR-FL or acts alone to compete with AR-FL binding at those sites remains to be determined. More importantly, our ChIP-seq data also indicate distinct AR-V7 binding sites that cannot be occupied by AR-FL under low-level androgen environments, despite these sites still containing AREs. A major difference is that AR-V7–specific chromatin sites are less enriched for the FOXA1 binding motif, and thus may be in more compact chromatin formations, supported by evidence for lower FOXA1 binding and ATAC signals at these sites. Therefore, a possible explanation for the differential AR-FL and AR-V7 chromatin binding activities is that they may interact with different coactivator/remodeler complexes. AR-V7 has a unique chromatin activity, in that it initially accesses the compact chromatin regions, possibly mediated by recruiting specific pioneer factor/chromatin remodeling complexes that can further facilitate chromatin opening. However, AR-FL binding is more dependent on the pioneer factor FOXA1, which may have low affinity for those AR-V7–specific chromatin regions due to the lack of perfect forkhead binding motifs. Overall, our data suggest that AR-V7 may have a distinct capability to access many cryptic AREs in CRPC cells and thus activate them to promote the expression of its unique targets. While we propose a model of AR-V7 activity independent of AR-FL, all our cell line models contain both AR-FL and AR-V7. Therefore, it remains to be determined whether this AR-V7 activity would occur solely in the absence of AR-FL, or whether it is just specifically regulated by AR-V7 in the presence of AR-FL.

Another major finding from this study is the discovery that the S81 phosphorylation of AR-V7 can enhance its pro-metastasis function by selectively altering the AR-V7–mediated transcription program. The phosphorylation of AR-FL S81 has been previously studied by us and other groups, and this modification may affect AR-FL protein degradation, subcellular localization, chromatin binding, and coactivator interactions (61–63, 65, 69, 70). However, it is not surprising that this phosphorylation may have a unique function in the AR-V7 protein due to its lack of a hinge region and LBD. Interestingly, this selective enhancement effect of the AR-V7 transcription program did not appear to associate with the alteration of its chromatin binding, since AR-V7 binding intensity at specific or common sites was not decreased by S81A mutation. Therefore, we propose a model in which S81 phosphorylation may enhance or weaken AR-V7’s interaction with its specific coregulators at AR-V7 unique sites, which are enriched for metastasis genes. This mechanistic model clearly requires further investigation. Overall, our study suggests that S81 phosphorylation in AR-V7 may serve as a possible biomarker for predicting the aggressiveness of CRPC and immunohistochemical staining of phosphorylated S81 has been successfully applied in patient samples (70). Moreover, the identification of such important posttranslational modifications of AR-V7 also allows us to identify critical druggable targets that are involved in the regulation of the AR-V7–mediated metastasis program. Therefore, we next determined whether treatments targeting S81 phosphorylation can be efficacious in suppressing metastasis. We particularly tested CDK9 inhibition in this study and showed that clinically tested CDK9 inhibitors can strongly suppress the expression of AR-V7–targeted genes and reduces metastasis. While it is highly unlikely that monotherapy with CDK9 inhibitors can completely suppress the metastatic progression of PCa after ARSi, our study provides a proof of concept for the future exploration of combination treatment strategies, particularly focusing on targeting AR-V7 phosphorylation early to prevent PCa cells from initiating more aggressive metastatic progress in bone. As more active and selective CDK9 inhibitors are being developed and entering the clinic, the preclinical findings in this study can likely accelerate the development and testing of such innovative AR-V7–targeting therapies in clinical trials and can be rapidly translated into patients.

## Methods

### Sex as a biological variable.

Our study exclusively examined male mice. It is unknown whether the findings are relevant for female mice.

### Cell lines.

LNCaP cells were cultured in RPMI with 10% fetal bovine serum (FBS) and C4-2 cells were cultured in RPMI with 2% FBS plus 8% charcoal-stripped FBS (CSS). Both cell lines were purchased from ATCC and authenticated periodically using short tandem repeat (STR) profiling. LNCaP and C4-2 stable cell lines overexpressing tetracycline-regulated AR-FL, AR-V7, and their S81A mutants were generated by lentiviral infection of the pLIX_403 tetracycline-inducible lentiviral vector with WT AR-FL/AR-V7 and S81A AR-FL/AR-V7 using Gateway Technology with Clonase II (Invitrogen, 12535-029). The S81A point mutation was generated using a QuickChange Lightning Site-Directed Mutagenesis Kit (Agilent Technologies, 210518) from WT AR-V7. LNCaP stable cell lines overexpressing cumate-regulated 3×FLAG-tagged ARv567es were generated by lentiviral infection of the Lenti-Cu3Flag-ARv567es vector. The culture conditions for LN-tet-Ctrl and LN-tet-SOX9 were described previously (27). All these stable cell lines were cultured with tetracycline-free FBS. CWR-22Rv1 cells were purchased from ATCC and cultured in RPMI with 10% FBS. LNCaP-95 cells were derived from parental LNCaP cells and cultured with 10% phenol red–free CSS. LuCaP 35CR cells were derived from a LuCaP 35CR PDX model and cultured with 10% FBS. All cell lines used in the zebrafish embryo metastasis assay were stably infected by GFP lentivirus (abm, LV006). For androgen stimulation assays, cells were grown to 50%–60% confluence in culture medium containing 5% CSS for 3 days and then treated with DHT. All cell lines were frequently tested for mycoplasma contamination using the MycoAlert mycoplasma detection kit (Lonza).

### Immunoblotting.

For immunoblotting, cells were lysed with RIPA buffer containing protease inhibitor cocktail and proteins were resolved by electrophoresis in precast gradient gels (Bio-Rad). The detailed antibody information is provided in Supplemental Methods.

### qRT-PCR.

RNA was isolated from cells with TRIzol reagent (Invitrogen). Quantitative real-time PCR was performed using Fast 1-step Mix (Thermo Fisher Scientific). PCR results were normalized to GAPDH. The detailed description for TaqMan primer/probe sets can be found in Supplemental Methods.

### RNA-seq analysis.

RNA from cell lines was extracted by using the RNeasy Kit (QIAGEN). The RNA-seq library was prepared using a TruSeq Stranded RNA LT Kit (Illumina). Sequencing was performed using an Illumina HiSeq 2500 or NextSeq 2000 Genome Analyzer. Differential gene expression analysis was performed using edgeR version 3.24.1 (https://bioconductor.org/packages/release/bioc/html/edgeR.html) with Benjamini-Hochberg FDR-adjusted *P* value of 0.05 and fold-change cutoff of 1.5 or 2. A more detailed description of further analyzing these data, including GSEA and KEGG analyses, is provided in Supplemental Methods.

### ChIP.

For the preparation of ChIP, cells were cross-linked with 1% paraformaldehyde, collected, and lysed with protease inhibitor cocktail–supplemented lysis buffer, and sonicated into 500- to 800-bp fragments for ChIP-qPCR by Bioruptor Sonicator (Diagenode), followed by immunoprecipitation with 4 μg of ChIP-grade antibodies. The ChIP-qPCR assays were then carried out with replicates and normalized to input DNA using SYBR Green Master Mix with the AR-V7 binding sites listed in Supplemental Methods. Antibodies and primers are listed in Supplemental Methods.

### ChIP-seq and ATAC-seq analyses.

DNA samples for ChIP-seq were prepared as described above and sonicated into 200- to 300-bp fragments. For the preparation of ATAC-seq, nuclei were treated with transposase using the Illumina Tagment DNA TDE1 Enzyme and Buffer Kit, and DNA samples were cleaned immediately with a Qiagen QIAquick Purification Kit and PCR-amplified by NEBNext High-Fidelity 2× PCR Master Mix. ChIP-seq and ATAC-seq libraries were constructed using the SMARTer ThruPLEX DNA-Seq Prep Kit (Takara Bio). Next-generation sequencing (51 nt, single-end) was performed using an Illumina HiSeq 2500 Genome Analyzer. MACS3 (version 3.0.0.a6) (71) was used to evaluate the significance of enriched ChIP-seq regions. A more detailed description of analyzing these data is provided in Supplemental Methods.

### Invasion assay.

Invasion assays were performed with Corning BioCoat Matrigel Invasion Chambers (354480, Corning). Per the manufacturer’s protocol, in brief, the same number of cells were seeded in the premoisturized upper chamber with serum-free medium, and the lower chamber was filled with medium containing 10% FBS as the chemoattractant. After 3 days, noninvading cells were removed by using a cotton swab, and the invaded cells were fixed with 100% methanol and then stained with Giemsa staining solution (Thermo Fisher Scientific). All experiments were done with biological triplicates and images were acquired with an EVOS auto fluorescence microscope.

### Zebrafish metastasis assay.

Adult AB WT zebrafish were crossed and the embryos were collected at approximately 4 hours after fertilization. These were raised until 2 days after fertilization, at which point they were treated with Pronase (10 mg/mL) for dechlorination. All injections were performed from the resultant larvae in accordance with the institutional approved protocol. The larvae were anesthetized with Tricaine before injecting with approximately 100 GFP-tagged cells targeting the perivitelline space. These injected embryos were then imaged with fluorescence microscopy for invasion within an hour after the injection. Embryos exhibiting positive circulation signals were classified as “invaded.” The figures in our manuscript represent the proportion of invaded embryos relative to the total number injected. The significance of difference was determined by using Fisher’s exact test or χ^2^ test, depending on sample size.

### Mouse xenografts.

NSG-SCID mice (Jackson Laboratory) were maintained and propagated in the Vivarium of Van Andel Institute (VAI) or the Department of Laboratory Animal Research, College of Medicine and Life Sciences at the University of Toledo. Male mice were castrated at approximately 4–5 weeks old and randomized into different groups for the injections with PCa cells or with PBS as a negative control. For using doxycycline-inducible stable cells, the injected mice were further randomized into 2 groups and fed with or without doxycycline-supplemented water (1 g/L, Takara Bio, 631311) until the end of experiments. For intratibial injection, 1 million cells in 10 μL of PBS were injected into the left and right mouse tibiae, as described previously (72, 73). Bone lesions and tumor growth were monitored weekly by radiographic imaging using a Faxitron x-ray machine at VAI and Xpert80 x-ray machine (CUBTEC Scientific) at the University of Toledo. The first x-ray image for each mouse was acquired immediately before intratibial injection as the starting point. The bone lesion areas and regions of interest (ROIs) were measured using MetaMorph (Molecular Devices) and analyzed using GraphPad Prism. Mice were euthanized at the end points and their left tibiae were harvested in 70% ethanol and subjected to microcomputed tomography (μCT) scanning and imaging using a μCT instrument (SKYSCAN 1172 at VAI and μCT35 at the University of Toledo). Data were further analyzed and processed as previously described (72). All measured variables in the tibiae with PCa were normalized to sham-injected tibiae that had undergone the same treatment and procedures. The right tibiae were fixed in 10% neutral buffered formalin, decalcified in 14% EDTA, and processed for paraffin embedding and sectioning. The tissue sections were further used for histology and immunohistology staining for detection of SOX9, detailed as previously described (72, 74, 75). The injected cell preparations, injections, imaging, tumor measurements, and bone lesion analyses were performed blinded.

### Statistics.

Data in bar graphs represent mean ± SD of at least 3 biological repeats. Bar-and-whisker plots show the median (line in box), 25th to 75th IQR (bounds of the box), 1.5× IQR (whiskers), and outliers (individual data points). Statistical analysis was performed using 2-sided Student’s *t* test by comparing treatment versus vehicle control or otherwise as indicated. Box-and-whisker plots of the signature score and gene expression were compared using Wilcoxon’s test for comparison between the 2 conditions. The mouse xenograft data were analyzed using 2-sided Student’s *t* test to compare the bone lesions between the control and treatment groups at different time points separately. The zebrafish metastasis data were analyzed using Fisher’s exact test or χ^2^ test, depending on sample size. All statistical analyses and visualization were performed with R (https://cran.r-project.org/) unless otherwise specified. A *P* value of less than 0.05 was considered to be statistically significant (*P < 0.05, **P <0.01, ***P < 0.001, ****P < 0.0001).

### Study approval.

The animal study protocols (zebrafish, mouse) were approved by the Institutional Animal Care and Use Committees (IACUC) at University of Massachusetts Boston, Van Andel Institute, and the University of Toledo.

### Data availability.

The RNA-seq, ChIP-seq, and ATAC-seq data generated from this study have been deposited in the NCBI Gene Expression Omnibus (GEO GSE221142). Values for all data points in graphs are reported in the Supporting Data Values file.

## Author contributions

CC, XL, KZ, DH, M Labaf, and YZ designed the study. DH, M Labaf, YZ, JO, SZ, KP, KO, KV, JSS, WH, M Li, M Liu, ZW, AB, SP, XY, SPB, PSN, SRP, SG, KRS, RL, MMS, GF, PJC, and BOW performed experiments and analyzed the results. SP, MJM, KZ, and JAM performed deep sequencing analyses. CC, DH, M Labaf, YZ, SRP, PSN, KZ, and XL wrote the manuscript. DH, M Labaf, and YZ contributed equally to the study: DH performed most molecular assays and zebrafish studies, M Labaf performed all the bioinformatical and statistical analyses, and YZ performed all the mouse studies. All authors discussed the results and commented on the manuscript. All data generated or analyzed during this study are included in this published article. Readers are welcome to comment on the online version of the paper.

## Supplementary Material

Supplemental data

Unedited blot and gel images

Supporting data values

## Version 1 04/30/2024

In-Press Preview

## Version 2 06/03/2024

Electronic publication
