# Supplementary material for: Androgen receptor splice variants drive castration-resistant prostate cancer metastasis by activating distinct transcriptional programs
Source: J Clin Invest. 2024 Apr 30;134(11):e168649. doi: 10.1172/JCI168649 (PMC11142739; doi:10.1172/JCI168649)
Supplement: Supplemental data [file jci-134-168649-s010.pdf]

SUPPLEMENTARY INFORMATION INVENTORY:

Figure S1, related to Figure 1.

Figure S2, related to Figure 2.

Figure S3, related to Figure 2.

Figure S4, related to Figure 2.

Figure S5, related to Figure 3.

Figure S6, related to Figure 4.

Figure S7, related to Figure 5.

Figure S8, related to Figure 6.

Figure S9, related to Figure 6.

Figure S10, related to Figure 8.

Figure S11, related to Figure 9.

Supplementary Materials and Methods

Supplementary References

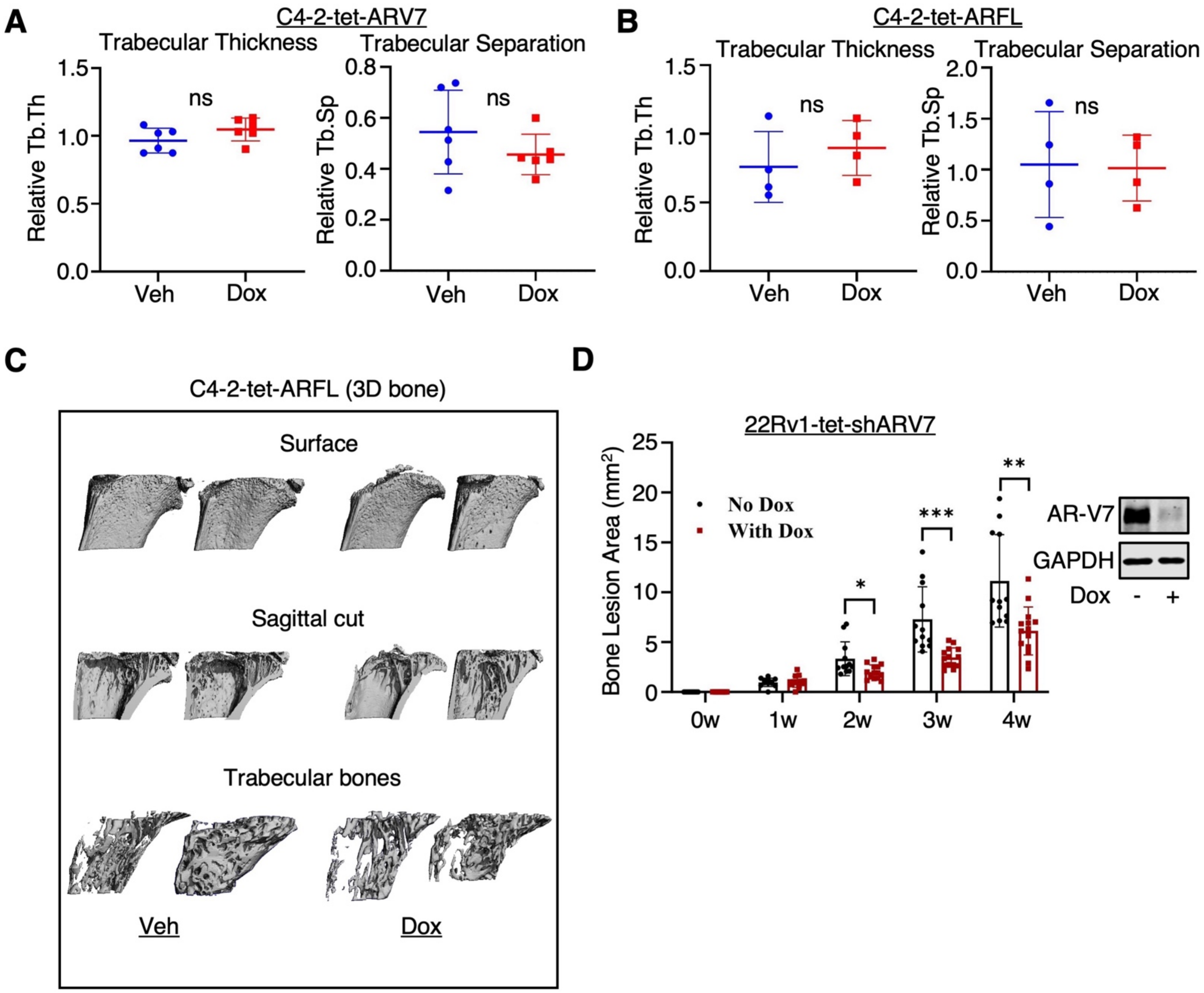

Supplementary Figure S1. AR-V7 overexpression accelerates bone metastasis

A, **B**, Normalized trabecular thickness and trabecular separation for the C4-2-tet-ARV7 (A) and C4-2-tet-ARFL (B) models. **C**, Bone surface views, sagittal cut views of bones, and structure views of trabecular bones, scanned by micro-CT and 3D reconstructed. **D**, 22Rv1 cells stably expressing doxycycline-regulated shRNA against AR-V7 were established. Immunoblotting was performed to confirm AR-V7 silencing (right panel). These stable cells were then injected into the tibias of castrated NSG male mice. Mice were then fed with diets supplemented with or without doxycycline. The bone lesion area was monitored and quantified (left panel). For the bar graph plot,

an unpaired two-sided *t*-test was used to determine statistical significance (* $P<0.05$, ** $P<0.01$, *** $P<0.001$, **** $P<0.0001$). Data are represented as mean ± standard deviation.

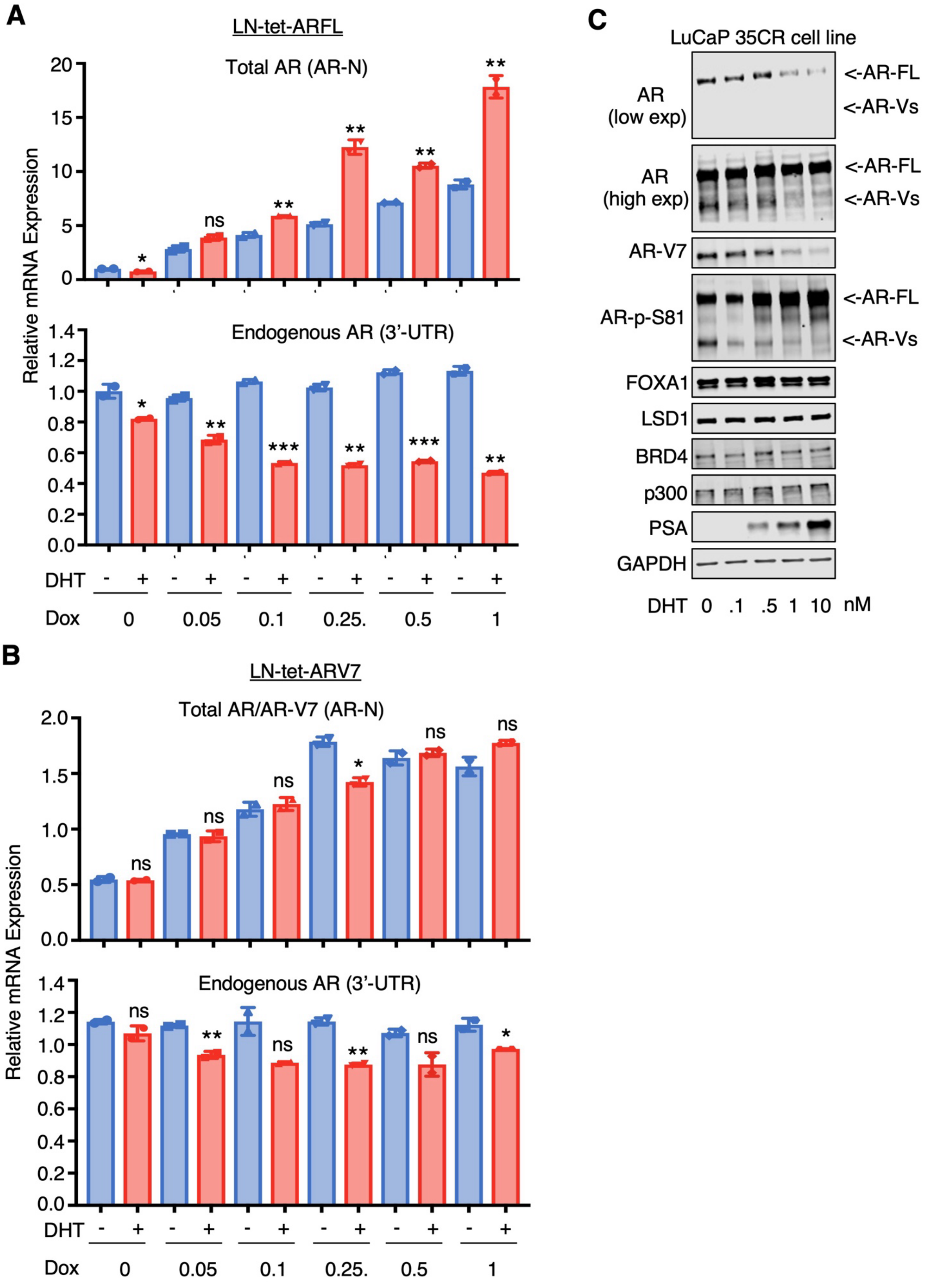
A
LN-tet-ARFL
Total AR (AR-N)
Endogenous AR (3'-UTR)
Relative mRNA Expression
DHT
Dox
0
0.05
0.1
0.25.
0.5
1
B
LN-tet-ARV7
Total AR/AR-V7 (AR-N)
Endogenous AR (3'-UTR)
Relative mRNA Expression
C
LuCaP 35CR cell line
AR (low exp)
AR (high exp)
AR-V7
AR-p-S81
FOXA1
LSD1
BRD4
p300
PSA
GAPDH
<-AR-FL
<-AR-Vs
DHT 0 .1 .5 1 10 nM

Supplementary Figure S2. Establishment of LN-tet-ARFL, LN-tet-ARV7, and 35CR cell lines

A, **B**, qRT-PCR for total AR and endogenous AR in LN-tet-ARFL (A) and LN-tet-ARV7 (B) cell lines. Cell were treated with doxycycline for 2d to induce AR overexpression, followed by 10nM DHT treatment for 24h in 5%CSS medium. **C**, 35CR cells were derived from the LuCaP 35CR PDX model. Immunoblotting was performed on cells stimulated with 0-10nM DHT for 24h to characterize the protein expression profile in this cell line model. All data are represented as mean ± standard deviation. For the bar graph plot, an unpaired two-sided *t*-test was used to determine statistical significance (* P<0.05, ** P<0.01, *** P<0.001, **** P<0.0001). Data are represented as mean ± standard deviation.

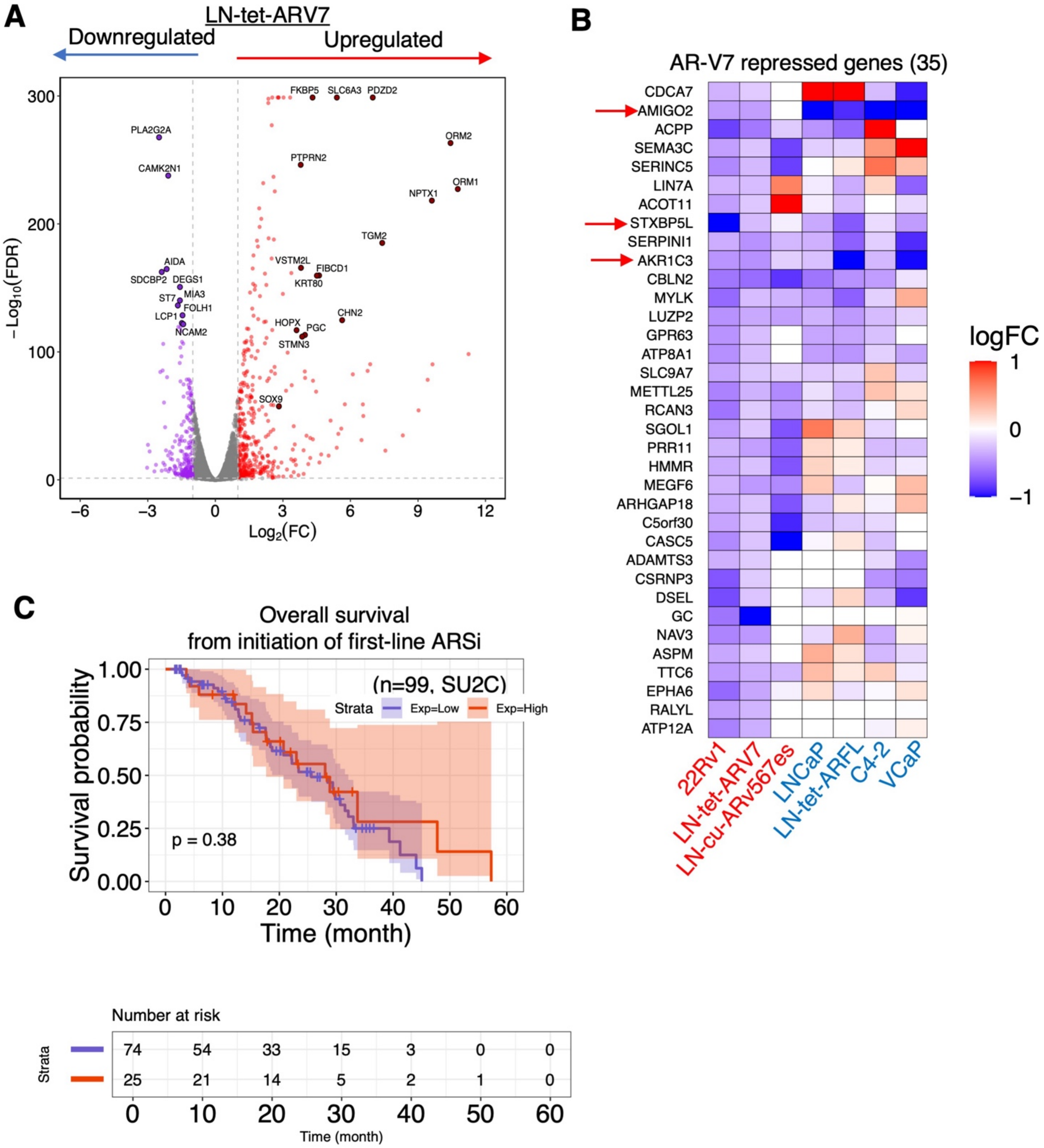

Supplementary Figure S3. Identification of genes repressed by AR-V7

A, Differential gene expression analysis was conducted in the LN-tet-ARV7 model treated with or without doxycycline treatment. Genes upregulated (red) or downregulated (blue) by AR-V7 were presented in a volcano plot. **B**, A 35-gene signature representing genes repressed by AR-V7 was identified in both LN-tet-AR-V7 and

22Rv1 cells. Heatmap illustrate the expression profiles of these genes in 22RV1, LN-tet-ARV7, or LN-cu-ARv567es cells under conditions of AR-V induction or silencing, and in LNCaP, LN-tet-ARFL (with doxycycline), C4-2, and VCaP cells with or without DHT treatment. **C**, Kaplan-Meier survival analysis was performed to assess overall survival from the initiation of first-line ARSi in mCRPC patients (SU2C cohort, N=99), comparing top 25 percentile of median score expression (red, N=25) versus lower 75 percentile (blue, N=74). *P*-value was calculated using the log-rank test from the score test.

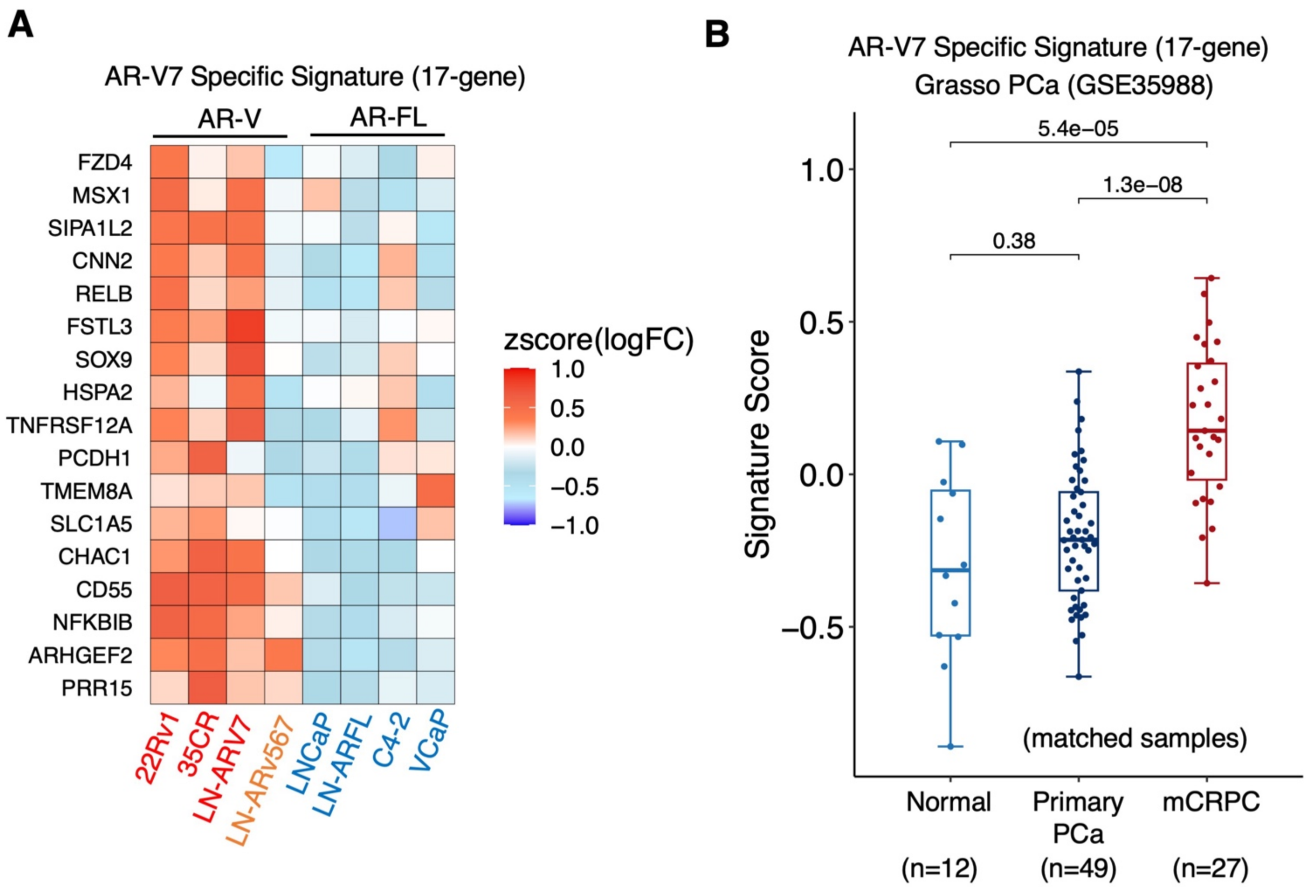

Supplementary Figure S4. Increase of AR-V7 specific signature in mCRPC

A, Heatmap views demonstrate the expression of the 17-gene signature specific to AR-V7 targets. **B**, Box plots for the levels of the AR-V7 target signature in a public PCa dataset (Grasso PCa, GSE35988, N=88) (1). Statistical analyses were conducted using unpaired nonparametric two-sample Wilcoxon test, with Bonferroni correction for multiple comparisons.

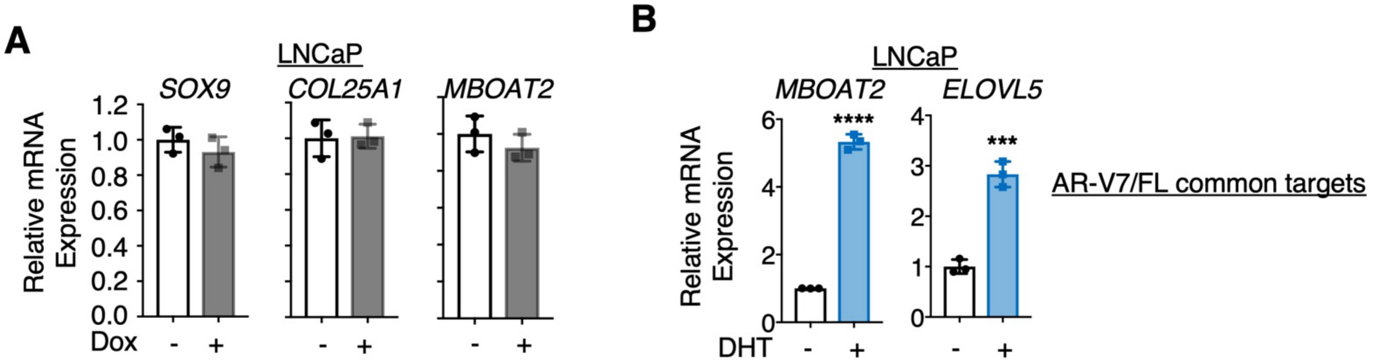

Supplementary Figure S5. Doxycycline treatment in LNCaP cells does not affect the expression of AR-V7 targets

A, qRT-PCR analysis of several AR-V7 targets in LNCaP cells, treated with or without doxycycline. **B**, qRT-PCR analysis of two AR-V7/AR-FL shared lipid synthesis targets in LNCaP cells, treated with or without 10nM DHT. For the bar graph plot, an unpaired two-sided *t*-test was used to determine statistical significance (* $P<0.05$, ** $P<0.01$, *** $P<0.001$, **** $P<0.0001$). Data are represented as mean ± standard deviation.

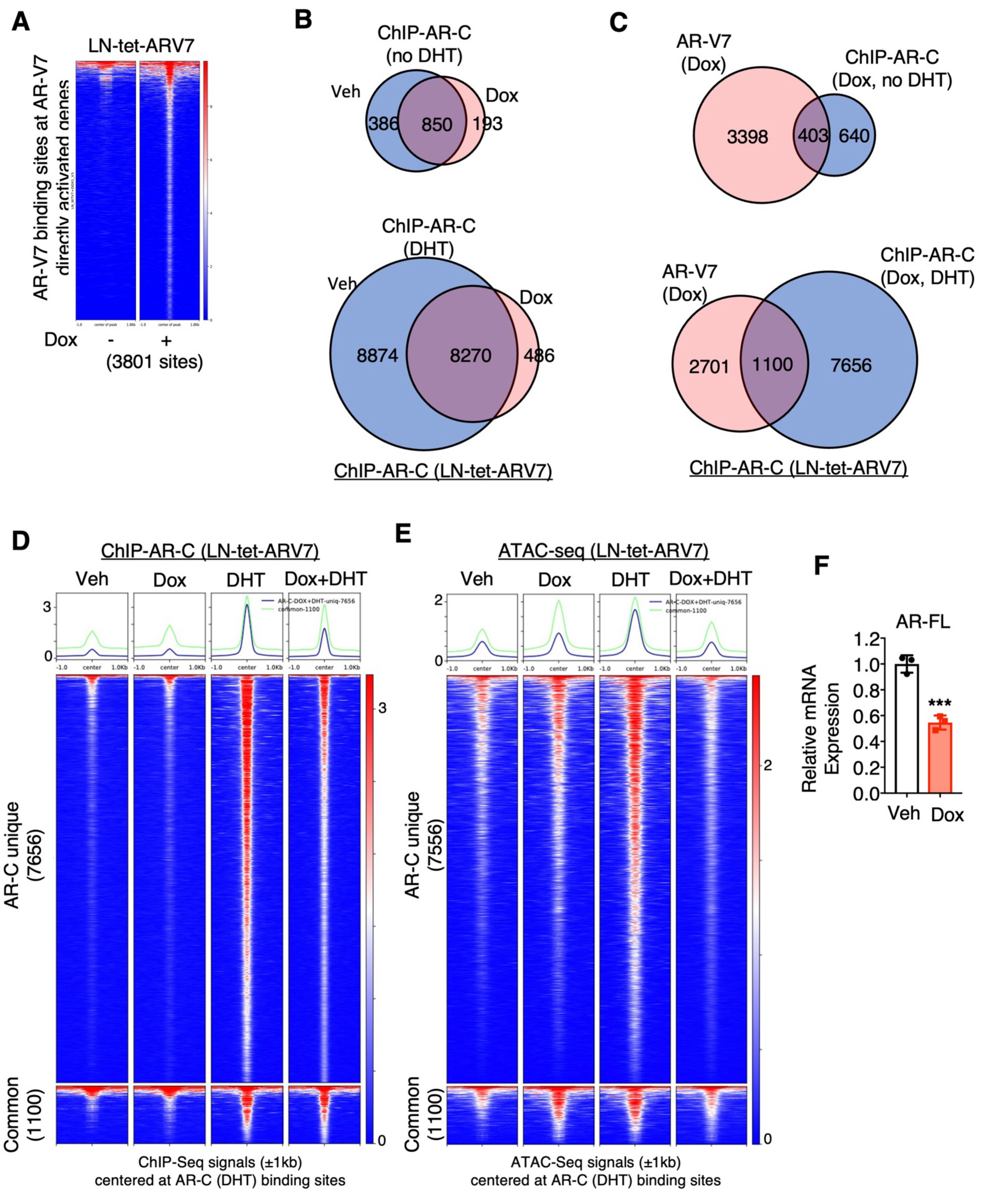
A
LN-tet-ARV7
AR-V7 binding sites at AR-V7 directly activated genes
Dox - +
(3801 sites)
B
ChIP-AR-C (no DHT)
Veh
Dox
386
850
193
ChIP-AR-C (DHT)
Veh
Dox
8874
8270
486
ChIP-AR-C (LN-tet-ARV7)
C
AR-V7 (Dox)
ChIP-AR-C (Dox, no DHT)
3398
403
640
AR-V7 (Dox)
ChIP-AR-C (Dox, DHT)
2701
1100
7656
ChIP-AR-C (LN-tet-ARV7)
D
ChIP-AR-C (LN-tet-ARV7)
Veh
Dox
DHT
Dox+DHT
AR-C unique (7656)
Common (1100)
ChIP-Seq signals (±1kb) centered at AR-C (DHT) binding sites
E
ATAC-seq (LN-tet-ARV7)
Veh
Dox
DHT
Dox+DHT
AR-C unique (7556)
Common (1100)
ATAC-Seq signals (±1kb) centered at AR-C (DHT) binding sites
F
AR-FL
Relative mRNA Expression
1.2
1.0
0.8
0.6
0.4
0.2
0.0
***
Veh
Dox

Supplementary Figure S6. AR-V7 expression represses the chromatin binding of endogenous AR-FL in LN-tet-ARV7 cells

A, Heatmap view of ChIP-seq analysis demonstrating V5-tagged AR-V7 binding in LN-tet-ARV7 cells (hormone-depleted), stimulated with or without doxycycline. **B**, Venn diagram showing chromatin binding peaks of AR-FL (detected using antibody against the C-terminal of AR) in LN-tet-ARV7 cells (hormone-depleted), under stimulation with or without doxycycline and with or without 10 nM DHT for 4h. **C**, Venn diagram illustrating the chromatin binding peaks of AR-FL with AR-V7 in these cells (under doxycycline treatment). **D**, **E**, Heatmap views for comparing the chromatin binding intensity of AR-FL (D) and ATAC-seq signals (E) at the unique sites versus the co-occupied sites. **F**, qRT-PCR analysis of AR-FL mRNA in LN-tet-ARV7 cells treated with or without doxycycline. For the bar graph plot, an unpaired two-sided *t*-test was used to determine statistical significance (* $P<0.05$, ** $P<0.01$, *** $P<0.001$, **** $P<0.0001$). Data are represented as mean ± standard deviation.

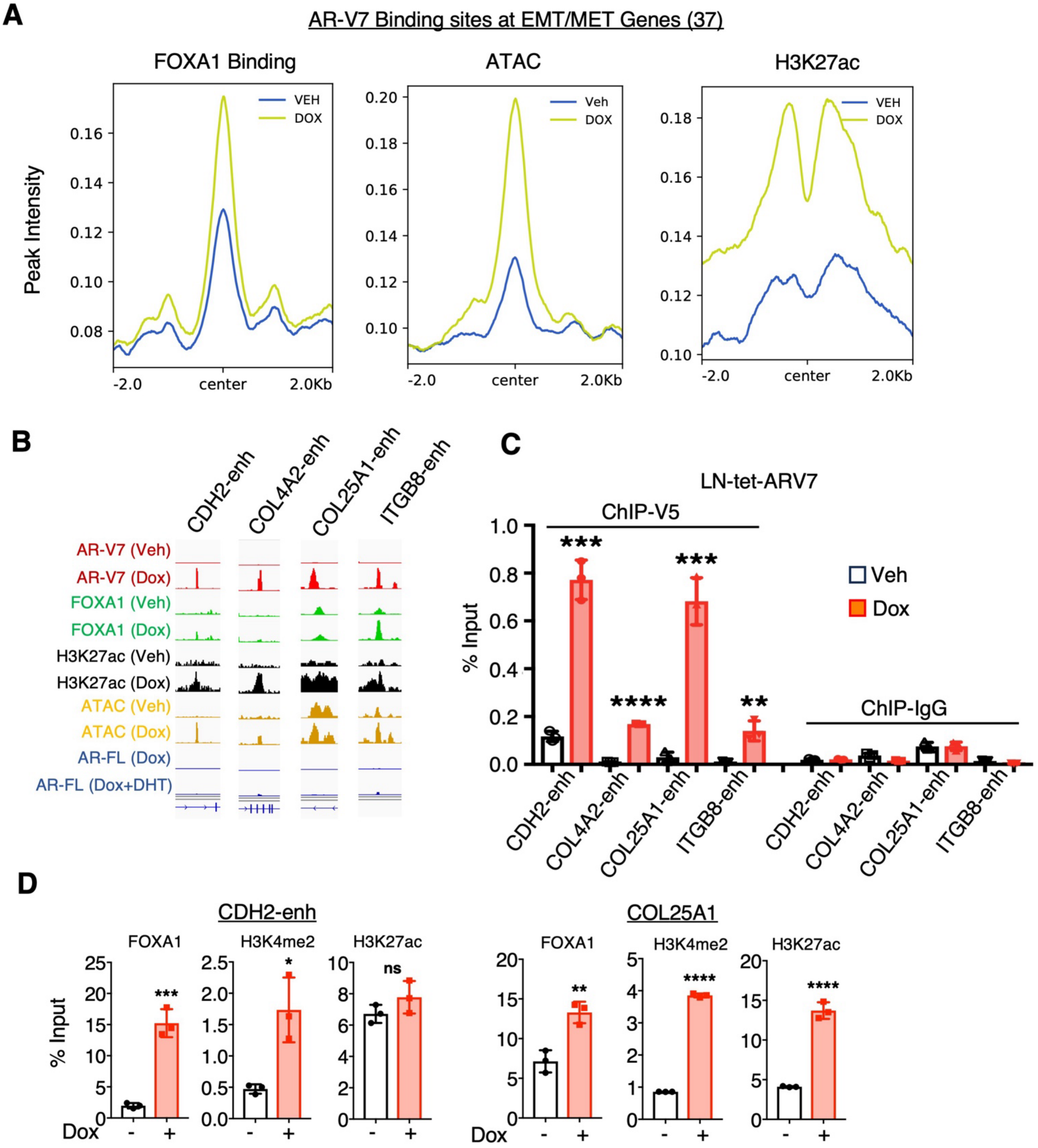

Supplementary Figure S7. Change of chromatin structure at AR-V7 specific binding sites

A, Analysis of average peak intensity of FOXA1 binding, ATAC signal, and H3K27ac levels at AR-V7 binding sites identified from its EMT/metastasis targets. **B**, Genome browser views for the bindings of indicated protein

at four AR-V7 specific sites. **C**, ChIP-qPCR analysis of V5 (V5-AR-V7) in LN-tet-ARV7 cells, stimulated with or without doxycycline, on several AR-V7 specific enhancer sites. **D**, ChIP-qPCR analysis of V5 (V5-AR-V7), FOXA1, H3K4me2, and H3K27ac in LN-tet-ARV7 cells, stimulated with or without doxycycline on AR-V7 specific enhancer sites. For the bar graph plot, an unpaired two-sided *t*-test was used to determine statistical significance (* P<0.05, ** P<0.01, *** P<0.001, **** P<0.0001). Data are represented as mean ± standard deviation.

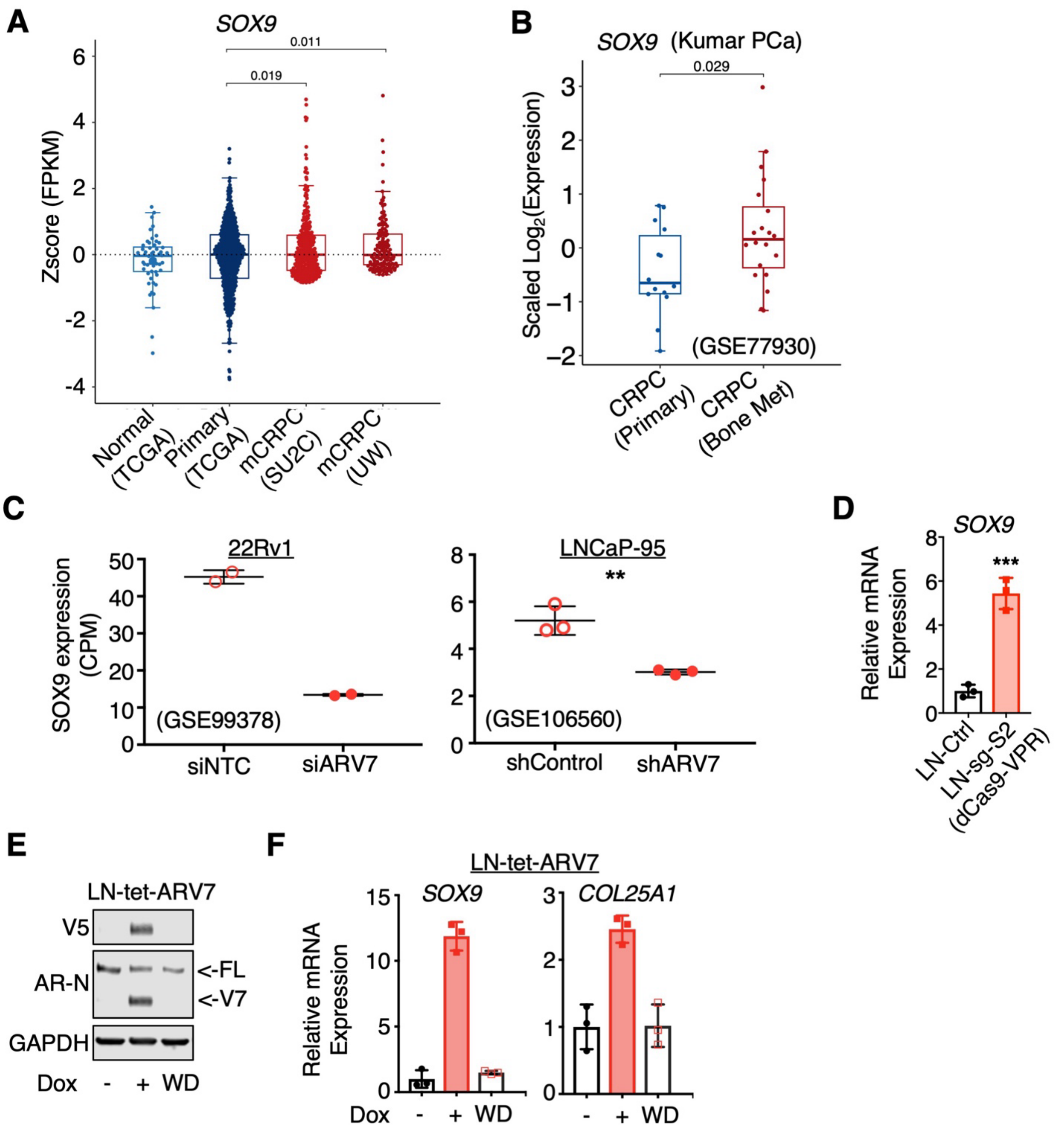

Supplementary Figure S8. SOX9 expression is upregulated in mCRPC and activated by AR-V7

A, Box blot showing SOX9 expression across TCGA (Normal N=52, primary PCa N=498), SU2C (N=266) and UW (N=138) patient datasets. **B**, Box blot of SOX9 expression in the Kumar PCa patient dataset (GSE77930) (2), comparing CRPC at primary sites (N=14) versus bone metastasis (N=20). **C**, Analysis of SOX9 expression in CRPC cells with AR-V7 alteration using public datasets (GSE99378 and GSE106560) (3, 4). **D**, qRT-PCR

analysis comparing mRNA expression of SOX9 in control cells versus a stable CRISPR cell line expressing dCas9-VPR (catalytically dead Cas9 fused with an activator complex) and sgRNA targeting the S2 site. **E**, **F**, Immunoblotting for V5 and N-terminal AR expression (E), and qRT-PCR analysis of AR-V7 targets (F) in LN-tet-ARV7 cells treated with a vehicle, doxycycline, and after doxycycline withdraw (replacing doxycycline-supplemented medium with fresh medium). Statistical analyses for (A, B, C) were conducted using unpaired nonparametric two-sample Wilcoxon test, with Bonferroni correction for multiple comparisons. For the bar graph plot, an unpaired two-sided *t*-test was used to determine statistical significance (* P<0.05, ** P<0.01, *** P<0.001, **** P<0.0001). Data are represented as mean ± standard deviation.

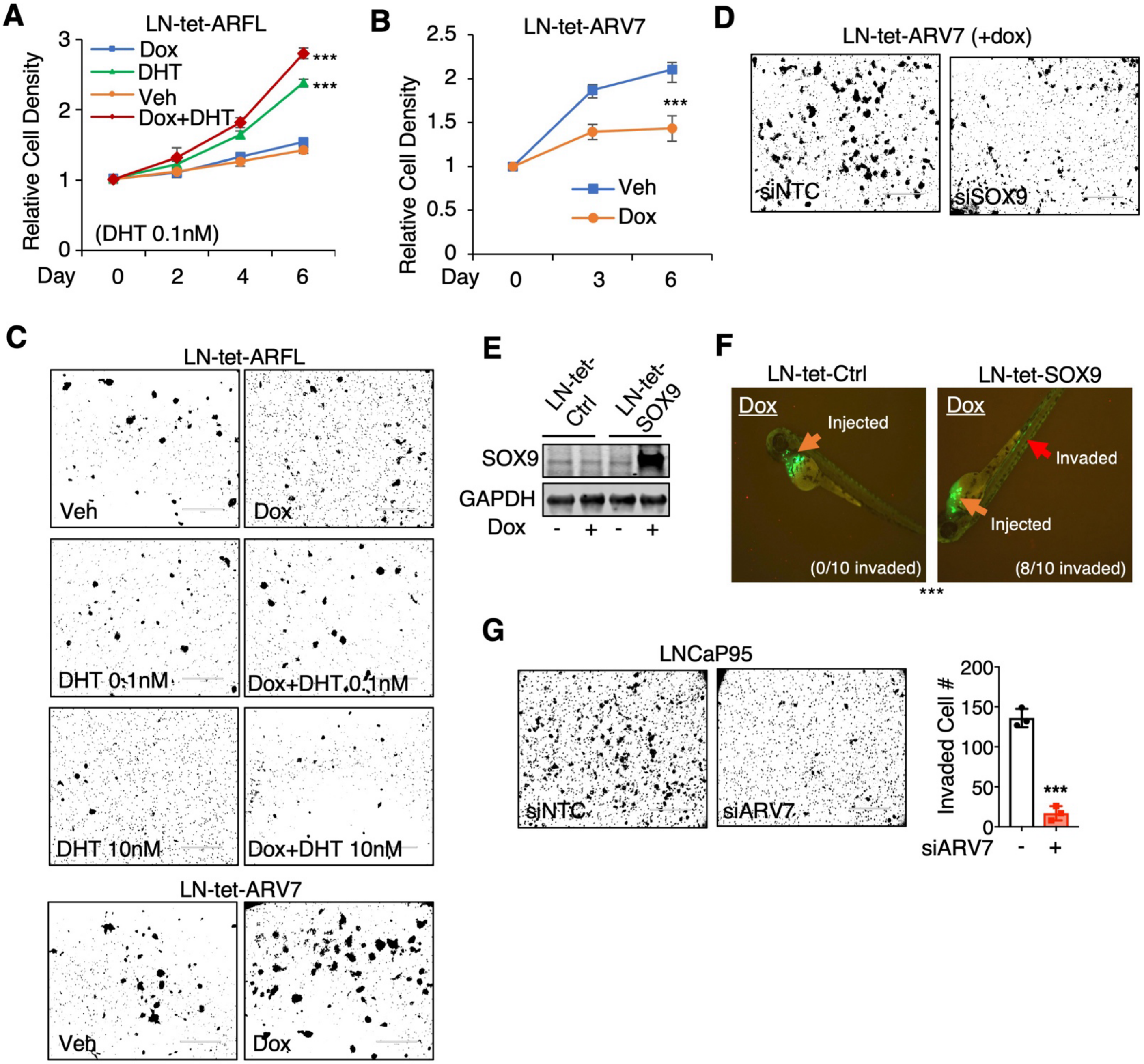

Supplementary Figure S9. AR-V7 induces PCa cell invasion but not proliferation

A, **B**, Cell proliferation assays in LN-tet-ARFL cells (A) and LN-tet-ARV7 cells (B), treated with or without doxycycline and with or without 0.1nM DHT for 6d. **C**, Cell invasion assays conducted in LN-tet-ARFL cells treated with or without doxycycline and with 0-10nM DHT, and in LN-tet-ARV7 cells treated with or without doxycycline. **D**, Cell invasion assay in doxycycline pre-treated LN-tet-ARV7 cells transfected with either siNTC

or siSOX9. **E**, **F**, Immunoblotting for SOX9 (E), and a zebrafish embryo metastasis assay (F) in LNCaP cells stably overexpressing an empty vector (LN-tet-Ctrl) or SOX9 (LN-tet-SOX9), treated with or without doxycycline. **G**, Cell invasion assay in LNCaP95 cells transfected with either siNTC or siARV7. For the bar graph plot, an unpaired two-sided *t*-test was used to determine statistical significance (* $P<0.05$, ** $P<0.01$, *** $P<0.001$, **** $P<0.0001$). Data are represented as mean ± standard deviation.

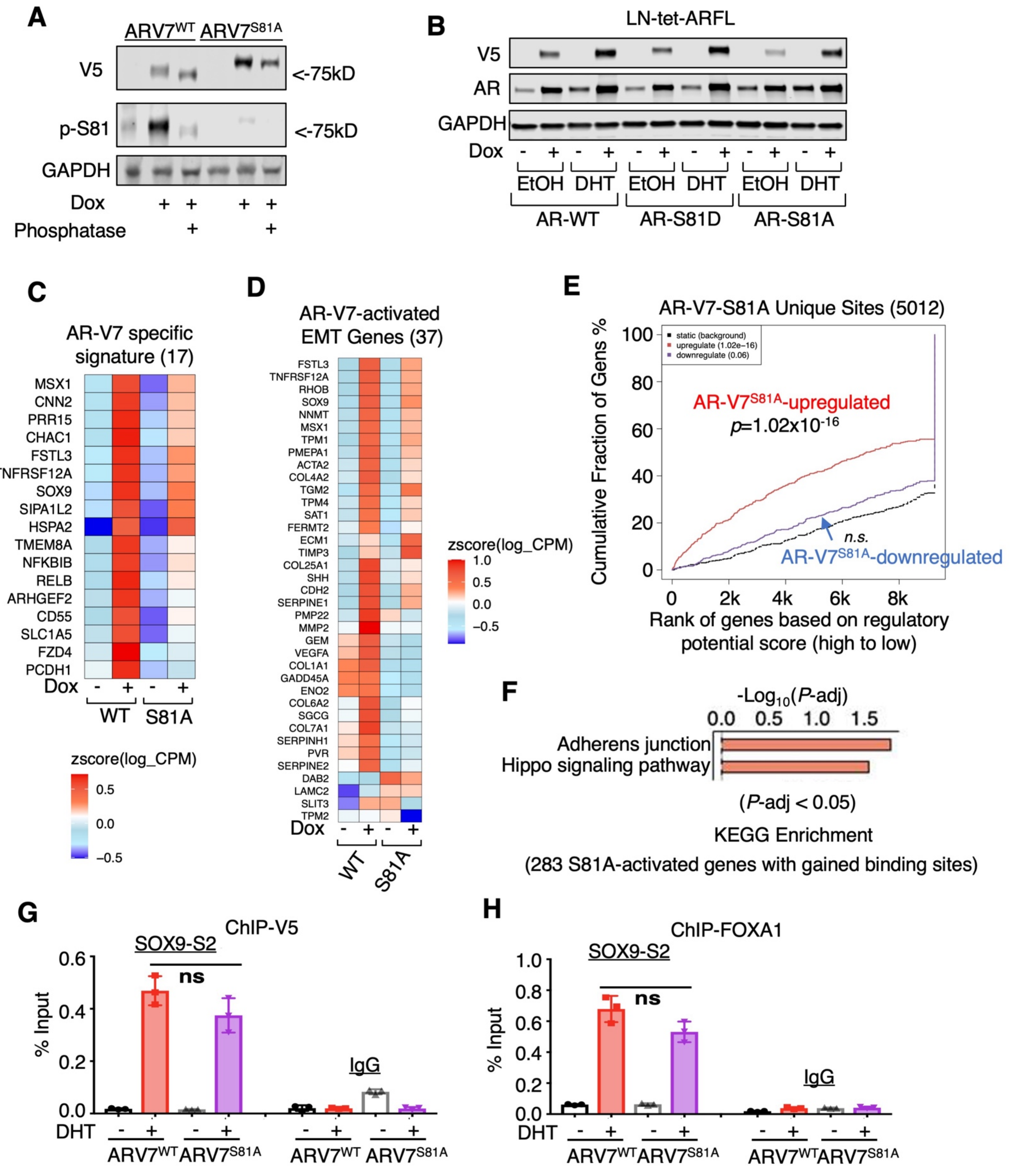
A
ARV7WT ARV7S81A
V5
p-S81
GAPDH
<-75kD
Dox
Phosphatase
B
LN-tet-ARFL
V5
AR
GAPDH
Dox
EtOH DHT EtOH DHT EtOH DHT
AR-WT AR-S81D AR-S81A
C
AR-V7 specific signature (17)
MSX1
CNN2
PRR15
CHAC1
FSTL3
TNFRSF12A
SOX9
SIPA1L2
HSPA2
TMEM8A
NFKBIB
RELB
ARHGEF2
CD55
SLC1A5
FZD4
PCDH1
WT S81A
zscore(log_CPM)
D
AR-V7-activated EMT Genes (37)
zscore(log_CPM)
E
AR-V7-S81A Unique Sites (5012)
static (background)
upregulate (1.02e−16)
downregulate (0.06)
AR-V7S81A-upregulated
p=1.02x10-16
n.s.
AR-V7S81A-downregulated
Cumulative Fraction of Gens %
Rank of genes based on regulatory potential score (high to low)
F
-Log10(P-adj)
Adherens junction
Hippo signaling pathway
(P-adj < 0.05)
KEGG Enrichment
(283 S81A-activated genes with gained binding sites)
G
ChIP-V5
SOX9-S2
ns
IgG
% Input
DHT
ARV7WT ARV7S81A ARV7WT ARV7S81A
H
ChIP-FOXA1
SOX9-S2
ns
IgG
% Input
DHT
ARV7WT ARV7S81A ARV7WT ARV7S81A

Supplementary Figure S10. Ser81 phosphorylation alters AR-V7-mediated transcription program

A, Immunoblotting analysis of indicated proteins in LN-tet-ARV7^{WT} and LN-tet-ARV7^{S81A} cells, treated with or without doxycycline and phosphatase (added to the protein extract). **B**, Immunoblotting for indicated proteins in LN-tet-ARFL^{WT}, LN-tet-ARFL^{S81D}, and LN-tet-ARFL^{S81A} cells, treated with or without doxycycline or 10nMDHT. **C**, **D**, Heatmap views displaying the identified AR-V7-specific signature © and AR-V7-activated EMT genes (D) in LN-tet-ARV7^{WT} and LN-tet-ARV7^{S81A} cells, treated with or without doxycycline. **E**, BETA analysis for the association of unique binding sites of AR-V7-S81A with AR-V7-S81A-regulated genes. **F**, KEGG pathway analysis for genes activated by AR-V7-S81A with nearby unique binding sites. **G**, **H**, ChIP-qPCR analysis for V5 (G) and FOXA1 (H) binding in LN-tet-ARV7 and LN-tet-ARV7^{S81A} cells, treated with or without doxycycline on the S2 site. For the bar graph plot, an unpaired two-sided *t*-test was used to determine statistical significance (* P<0.05, ** P<0.01, *** P<0.001, **** P<0.0001). Data are represented as mean ± standard deviation.

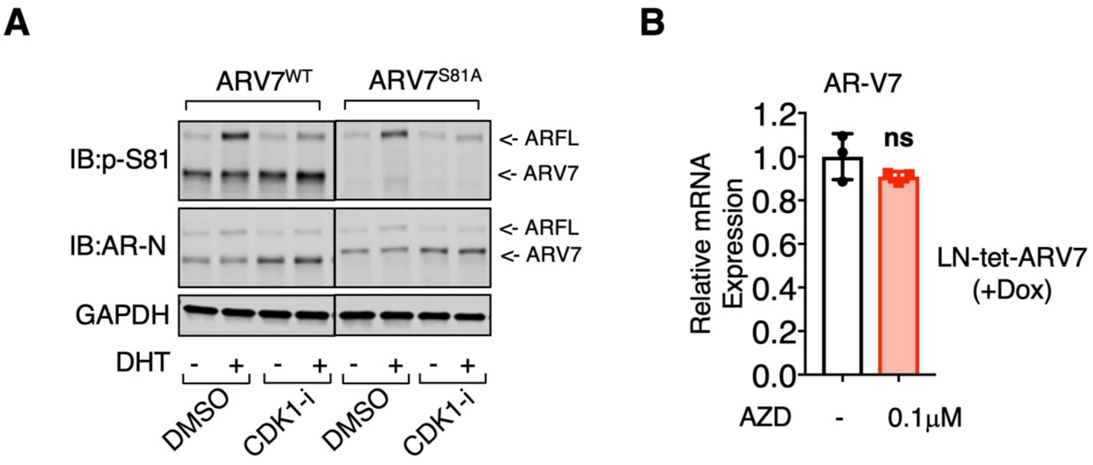

Supplementary Figure S11. CDK1 inhibitor treatment does not block Ser81 phosphorylation of AR-V7

A, Immunoblotting analysis of indicated proteins in LN-tet-ARV7^{WT} and LN-tet-ARV7^{S81A} cells stimulated with doxycycline and then treated with or without DHT or with CDK1 inhibitor (roscovitine, 10μM) for 24h. **B**, qRT-PCR analysis of AR-V7 in LN-tet-ARV7 cells, cultured under doxycycline and then treated with or without 0.1μM AZD4573 (24h). For the bar graph plot, an unpaired two-sided *t*-test was used to determine statistical significance (* $P<0.05$, ** $P<0.01$, *** $P<0.001$, **** $P<0.0001$). Data are represented as mean ± standard deviation.

SUPPLEMENTARY MATERIALS AND METHODS

CRISPR Activation Assay:

For CRISPR gene activation approach, LNCaP cells were stably infected with lentiviral dCas9-VPR (Dharmacon), which expresses catalytically deactivated Cas9 fused to VP64, p65, and Rta transcription activator complex (VPR), followed by blasticidin selection for 10 days. The established dCas9-VPR stable cells were then infected with the lentiviral sgRNA (5'-GGACACAATGTGATCATGAG-3', Addgene) against the S2 site (sgRNA-S2), followed by puromycin selection for 10 days.

Site-directed Mutagenesis:

LN-tet-ARV7^{S81A}, C4-2-tet-ARV7^{S81A}, LN-tet-ARFL^{S81A}, and LN-tet-ARFL^{S81D} cell line were generated by lentiviral infection of pLIX_403 with AR-V7^{S81A} (A->G, G->C), AR-FL^{S81A} (A->G, G->C), or AR-FL^{S81D} (A->G, G->A), which the point mutations were generated using QuickChange Lightning Site-Directed Mutagenesis Kit (cat# 210518, Agilent Technologies) from wild-type ARV7 plasmid. The primers for generating AR-V7^{S81A} or AR-FL^{S81A} are: forward, 5'-ctgcctgggggcagtctcttgctgctgctgc-3'; reverse, 5'-gcagcagcagcaagagactgcccccaggcag-3'. The primers for generating AR-FL^{S81D} are: forward, 5'-ctgcctggggtcagtctcttgctgctgctgc-3'; reverse, 5'-gcagcagcagcaagagactgaccccaggcag-3'.

RNA Interference: Short interfering RNAs (siRNA) targeting AR-V7 and non-targeting control (NTC) (D-001810-10-20) were purchased from Dharmacon (ON-TARGETplus). siARV7 sequence is: 5'-GTAGTTGTGAGTA-TCATGATT-3'. 22Rv1-tet-shARV7 cells were generated by stable lentiviral infection of 22Rv1 cells with shARV7-containing Tet-GFP-shAR-V7 tetracycline-inducible lentiviral vector. The short hairpin RNA sequence of shARV7 is: 5'-ccggGTAGTTGTGAGTATCATGActcgagTCATGATACTCACAACTACtttttg-3'. C4-2-tet-ARV7/shSOX9 cells were generated by the stable lentiviral infection of shSOX9 containing psi-LVRU6MH tetracycline-inducible lentiviral vector purchased from GeneCopoeia (HSH127361-LVRU6MH-a) with C4-2-tet-ARV7 cells. The SOX9 targeting sequence is: 5'-GCTCTGGAGACTTCTGAACGA-3'.

Signature Analysis:

The process to identify the AR-V7 specific signature (17-gene) is as follows: **1**) The common AR-V7-activated genes were identified by using the LN-tet-ARV7 model (overexpression model) with fold-change >= 1.5 and FDR <= 0.05 and 22Rv1/35CR models (silencing models) with fold-change >= 1.5 and FDR <= 0.05; **2**) These genes were then filtered by excluding AR-FL regulated genes in the LN-tet-ARFL model (10nM DHT versus vehicle, no doxycycline) and LN-tet-ARFL mode (0.1nM DHT versus vehicle, with doxycycline) with fold-change >= 2 and FDR <= 0.05. To identify the AR-V7 specifically activated EMT gene signature (37-gene), the hallmark EMT gene-set was retrieved from the Molecular Signature Database (MSigDB). The genes with a positive enrichment score from LN-tet-ARV7, 22Rv1 and 35CR models were selected. Then median fold-change of their expression with respect to their control of these three cell lines were calculated and the genes with top 75 percentile were chosen.

RNA-Seq Data Analysis: Transcriptome-sequencing reads were aligned to the human reference genome (GRCh37) using STAR (version2.5.3.a) (5). Gene expression counts were determined using featureCounts (version 1.6.2) (6) using protein-coding genes from Ensemble annotations (Ensembl.GRCh37.87). Differential gene expression analysis was performed using edgeR (version 3.24.1) with Benjamini-Hochberg false discovery rate (FDR)-adjusted *P*-value of 0.05 and fold-change cutoff of 1.5 or 2. The pre-ranked gene lists were used to conduct Gene Set Enrichment Analysis (GSEA) using the R package fgsea (version 1.18.022.0) (7) with msigdbr Hallmark gene sets (version 7.5.1). The top pathways with normalized enrichment scores (NES) ranked by adjusted *P*-value were plotted for visualization. The KEGG pathways analysis were performed using the R package gprofiler (version 0.2.1) (8). Significantly enriched pathways were determined using FDR *q*-value < 0.05 cutoff.

ChIP-seq and ATAC-seq Data Analysis: ChIP-seq and ATAC-seq fastq reads were mapped to the human reference genome (GRCh37) using BWA (version 0.7.5.a) (9). The aligned sam files were converted to bam format using Samtools (version 0.0.19) (10). MACS3 (version 3.0.0.a6) (11) was used to evaluate the significance of enriched ChIP-seq regions. The MACS bedgraph output files of signal per million reads were converted into bigwig files using UCSC tools (version 3.0.2) (11). The score per genomic region for each bigwig file was calculated using computeMatrix with reference point mode and the heatmap for the score associated with each genomic region was plotted with plotHeatmap from deepTools (version 3.0.2) (12). The ChIP-seq peak annotation and peak interval analysis were performed using the ChIPpeakAnno R package (version 3.26.4). Motif enrichment analysis was performed in SeqPos in Galaxy/Cistrome using the top 5000 narrowPeaks sorted by $\log_{10}$(*q*-value) (13). Integration of ChIP-seq transcription factor chromatin binding with differential gene expression data were performed by Binding and Expression Target Analysis (BETA) (version 1.0.7) (14)

Clinical Data Analysis:

For SU2C mCRPC, UW mCRPC, TCGA benign/primary PCa, and the Grasso PCa (GSE35988) patient data sets, the activity score was computed based on the 17-gene AR-V7 specific target signature. To determine the enrichment of AR-V7 specific target gene-set (17-gene) over the sample population, we applied the non-parametric and unsupervised gene set enrichment analysis using GSVA (version 1.46.0) R package applying ssgsea method. The z-score relative to all the samples for patient datasets were used as input. For SU2C (polyA+), UW and TCGA datasets, the log2((FPKM)) gene expression values from all tumors for each data set were converted to z-score, $z=(x- \mu)/\sigma$. Here μ is the average and σ is the standard deviation of log2(FPKM) across all the tumors for a given gene.

We performed the Kaplan-Meier survival analysis on the SU2C cohort. The z-score of mRNA expression of SU2C polyA+ (n = 266) and Capture (n = 208) were used as input. The data was filtered to include patient who received an ARSi treatment (abiraterone, enzalutamide, or apalutamide) prior to taxane, or the combination

therapy with another agent in the clinical trial. The expression profile of tissue samples was generated before the start of therapy or within 90 days after starting first-line therapy. For remaining tumor samples (n = 99), the median expression score was calculated using the median of 17 AR-V7 genes. The Kaplan-Meier survival was conducted on comparing top 25 percentile of median score expression with AR-V7 (SRPM) expression >0 (n = 25) versus lower 75 percentile with AR-V7 (SRPM) expression <= 0 (n = 74). Survival curves were calculated by survfit R function (version 3.2-0) and the statistical significance between two groups were assessed by log-rank test. Results were visualized using the survminer R package (version 0.4.9).

The CAI_BONE_MET gene-set were generated from the GSE32269 public data (15) with 22 primary PCa (hormone-dependent) and 29 bone metastatic PCa (CRPC) (16). We retrieved the gene expression data and applied the regression model with L1- regularization (Lasso Regression) on the 100 bootstrap samples. The predicted genes were ranked by the number of the times they were picked by bootstrap regularization. Top 50 genes were selected for further downstream analysis. We then used a GSEA software gene-set preprocess to further select 44 genes as the final CAI_BONE_MET gene-set. The expression values for barplots and heatmap were normalized by [x-median(x)]/[max(x)-min(x)].

Antibodies for Immunoblotting: Anti-V5 (Abcam, cat# ab9116), anti-Flag (Sigma, cat# F3165), anti-AR (Millipore, cat# 06-680), anti-ARV7 (RevMAb Biosciences, cat# 31-1109-00), anti-SOX9 (Bio-Rad, cat# VMA00211), anti-phospho-ARSer81 (Millipore, cat# 04-078) and anti-GAPDH (Abcam, cat# ab8245) were used as primary antibodies.

Antibodies for ChIP: ChIP grade antibodies used in this study are anti-V5 (Thermo Scientific, cat# R960-25), anti-AR-N-terminus (Abcam, cat# ab108341), anti-AR-C-terminus (Abcam, cat# ab52615), anti-FOXA1 (Abcam, cat# ab23738,), H3K27ac (Abcam, cat# ab4729), anti-H3K4me2 (Millipore, cat# 07-030) or Rabbit/Mouse IgG (Millipore).

Quantitative Real-time RT-PCR Primers: All TaqMan primer/probe sets were predesigned and purchased from Thermo Fisher Scientific: *SOX9* (Hs01001343_g1), *SHH* (Hs00179843_m1), *CDH2* (Hs00983056_m1), *COL4A2* (Hs05006309_m1), *COL25A1* (Hs00930851_m1), *ITGB8* (Hs00174456_m1), *MBOAT2* (Hs00294102_m1), *ELOVL5* (Hs01094704_m1).

ChIP-qPCR Primers:

SOX9-S2:

Forward, 5'-AACTCCCAGCACCCACAGTAC-3';

Reverse, 5'-GGAAGGGTCTGTCCCTGCTTT-3'.

CDH2-Enh:

Forward, 5'-ACAAGAGTTTGATGGACAAAAAT-3';

Reverse, 5'-GGTGACATTCTGTACCACATT-3'.

COL4A2-Enh:

Forward, 5'-AGCACAAGCAGGCCAATCTA-3';

Reverse, 5'-AGCAATGGAAGAACAGTGTG-3'.

COL25A1-Enh:

Forward, 5'-TGCAACTTCAAACTAAGGCACA-3';

Reverse, 5'-AGTGACTGAGGGAGTGATGC-3'.

ITGB8-Enh:

Forward, 5'-AGTTATAGCCCAAGCCTTCTCT-3';

Reverse, 5'-AGCACGTTTCCTCCTAGCAC-3'.

SUPPLEMTARY REFERENCES

1. Grasso CS, Wu YM, Robinson DR, Cao X, Dhanasekaran SM, Khan AP, et al. The mutational landscape of lethal castration-resistant prostate cancer. *Nature.* 2012;487(7406):239-43.
2. Kumar A, Coleman I, Morrissey C, Zhang X, True LD, Gulati R, et al. Substantial interindividual and limited intraindividual genomic diversity among tumors from men with metastatic prostate cancer. *Nat Med.* 2016;22(4):369-78.
3. Chen Z, Wu D, Thomas-Ahner JM, Lu C, Zhao P, Zhang Q, et al. Diverse AR-V7 cistromes in castration-resistant prostate cancer are governed by HoxB13. *Proc Natl Acad Sci U S A.* 2018;115(26):6810-5.
4. Cato L, de Tribolet-Hardy J, Lee I, Rottenberg JT, Coleman I, Melchers D, et al. ARv7 Represses Tumor-Suppressor Genes in Castration-Resistant Prostate Cancer. *Cancer Cell.* 2019;35(3):401-13 e6.
5. Dobin A, Davis CA, Schlesinger F, Drenkow J, Zaleski C, Jha S, et al. STAR: ultrafast universal RNA-seq aligner. *Bioinformatics.* 2013;29(1):15-21.
6. Liao Y, Smyth GK, and Shi W. featureCounts: an efficient general purpose program for assigning sequence reads to genomic features. *Bioinformatics.* 2014;30(7):923-30.
7. Subramanian A, Tamayo P, Mootha VK, Mukherjee S, Ebert BL, Gillette MA, et al. Gene set enrichment analysis: a knowledge-based approach for interpreting genome-wide expression profiles. *Proc Natl Acad Sci U S A.* 2005;102(43):15545-50.
8. Reimand J, Kull M, Peterson H, Hansen J, and Vilo J. g:Profiler--a web-based toolset for functional profiling of gene lists from large-scale experiments. *Nucleic Acids Res.* 2007;35(Web Server issue):W193-200.
9. Li H, and Durbin R. Fast and accurate long-read alignment with Burrows-Wheeler transform. *Bioinformatics.* 2010;26(5):589-95.
10. Li H, Handsaker B, Wysoker A, Fennell T, Ruan J, Homer N, et al. The Sequence Alignment/Map format and SAMtools. *Bioinformatics.* 2009;25(16):2078-9.

11. Zhang Y, Liu T, Meyer CA, Eeckhoute J, Johnson DS, Bernstein BE, et al. Model-based analysis of ChIP-Seq (MACS). *Genome Biol.* 2008;9(9):R137.

12. Ramirez F, Ryan DP, Gruning B, Bhardwaj V, Kilpert F, Richter AS, et al. deepTools2: a next generation web server for deep-sequencing data analysis. *Nucleic Acids Res.* 2016;44(W1):W160-5.

13. Liu T, Ortiz JA, Taing L, Meyer CA, Lee B, Zhang Y, et al. Cistrome: an integrative platform for transcriptional regulation studies. *Genome Biol.* 2011;12(8):R83.

14. Wang S, Sun H, Ma J, Zang C, Wang C, Wang J, et al. Target analysis by integration of transcriptome and ChIP-seq data with BETA. *Nat Protoc.* 2013;8(12):2502-15.

15. Cai C, Wang H, He HH, Chen S, He L, Ma F, et al. ERG induces androgen receptor-mediated regulation of SOX9 in prostate cancer. *J Clin Invest.* 2013;123(3):1109-22.

16. Stanbrough M, Bubley GJ, Ross K, Golub TR, Rubin MA, Penning TM, et al. Increased expression of genes converting adrenal androgens to testosterone in androgen-independent prostate cancer. *Cancer Res.* 2006;66(5):2815-25.
