# Supplementary material for: Androgen receptor splice variants drive castration-resistant prostate cancer metastasis by activating distinct transcriptional programs
Source: J Clin Invest. 2024 Apr 30;134(11):e168649. doi: 10.1172/JCI168649 (PMC11142739; doi:10.1172/JCI168649)
Supplement: Unedited blot and gel images [file jci-134-168649-s011.pdf]

Fig1A

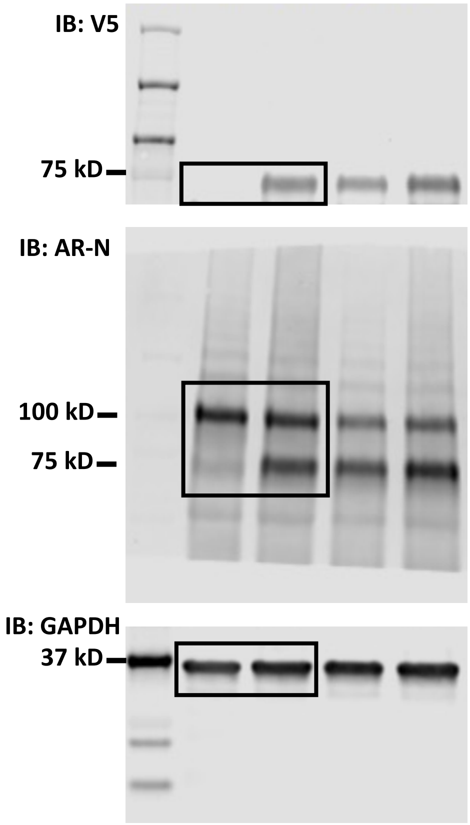

Fig1J

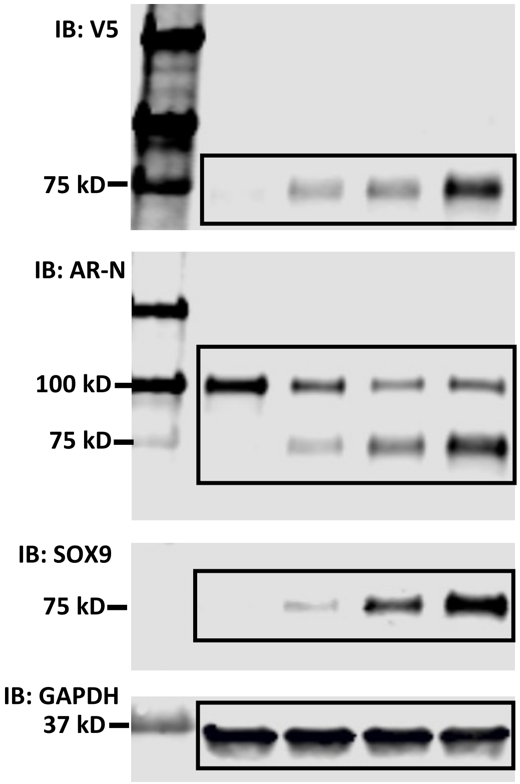

Fig1B

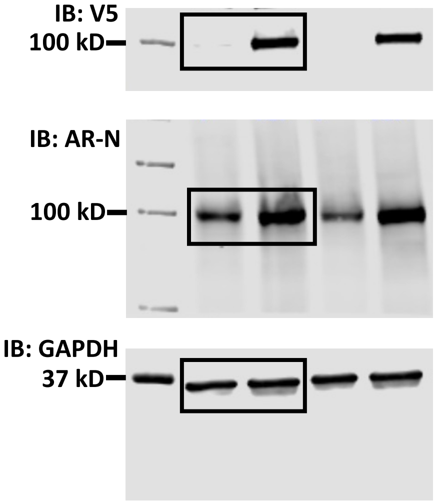

Fig1K

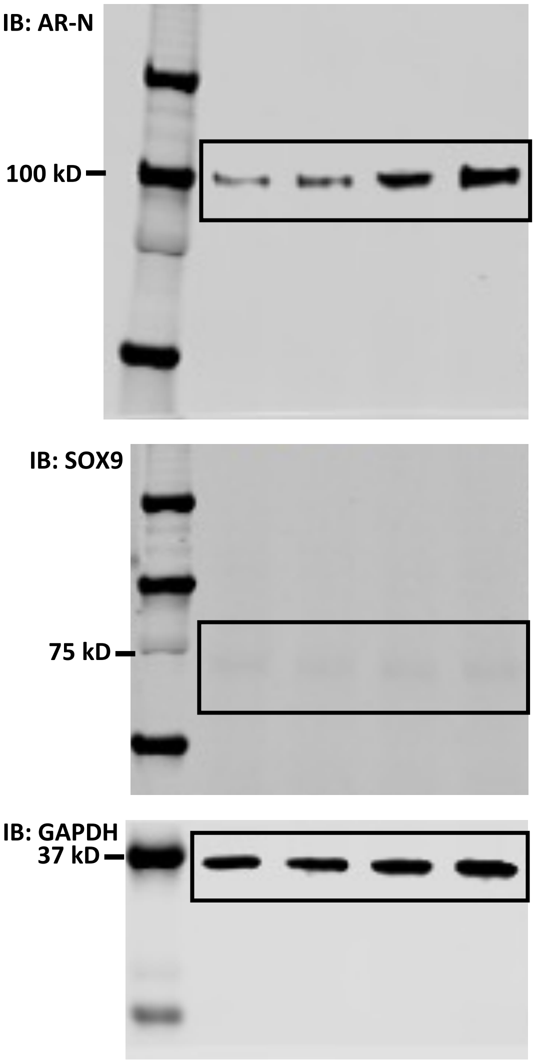
IB: AR-N
100 kD
IB: SOX9
75 kD
IB: GAPDH
37 kD

Fig2A

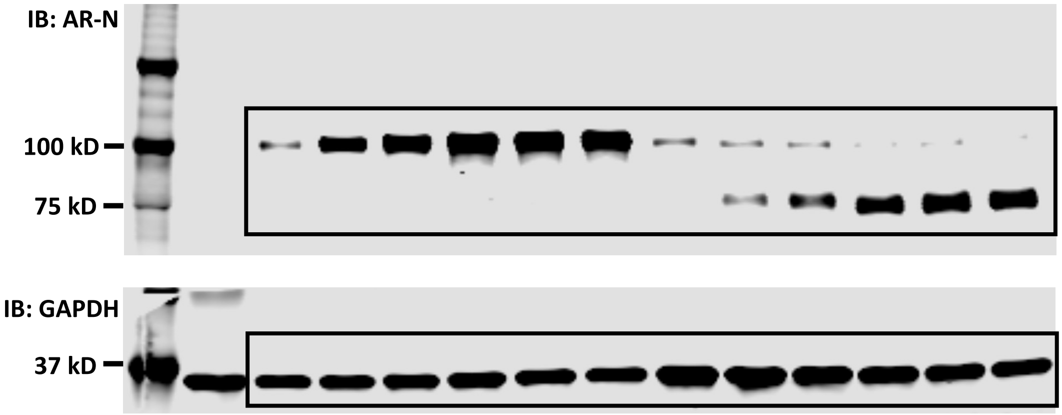
IB: AR-N
100 kD
75 kD
IB: GAPDH
37 kD

Fig2B

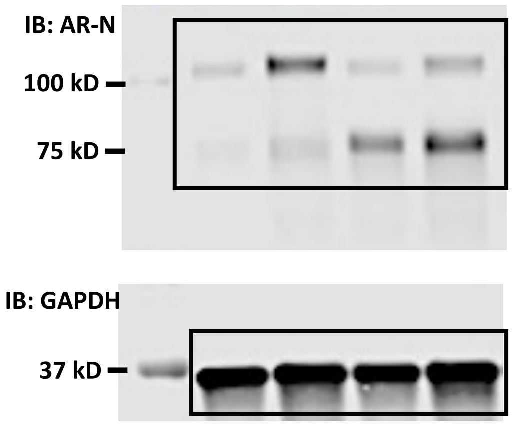

Fig2C

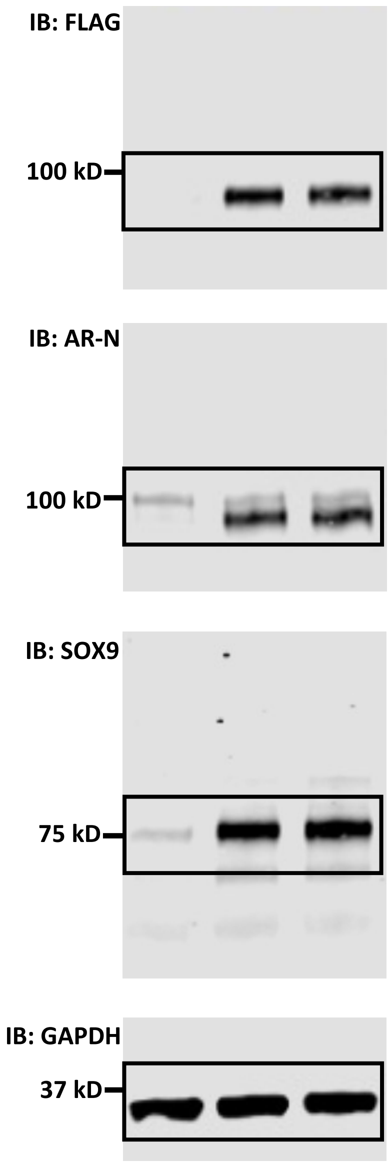

Fig2D

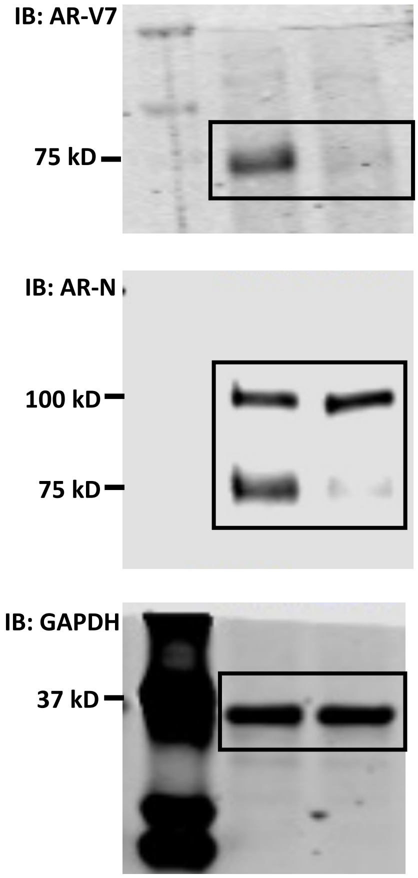

Fig2E

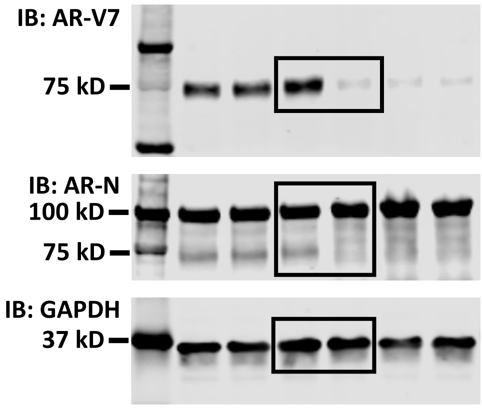

Fig6F

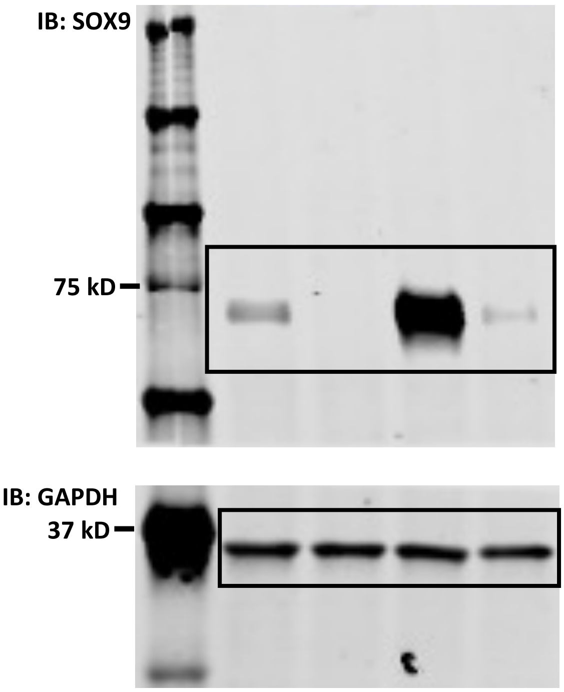

Fig6J

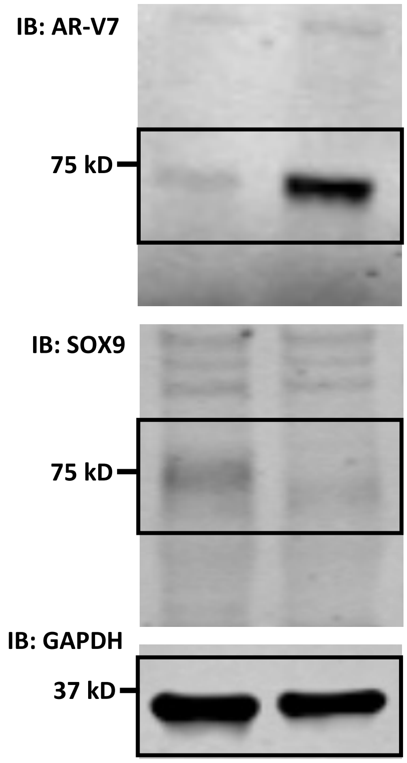

Fig6H

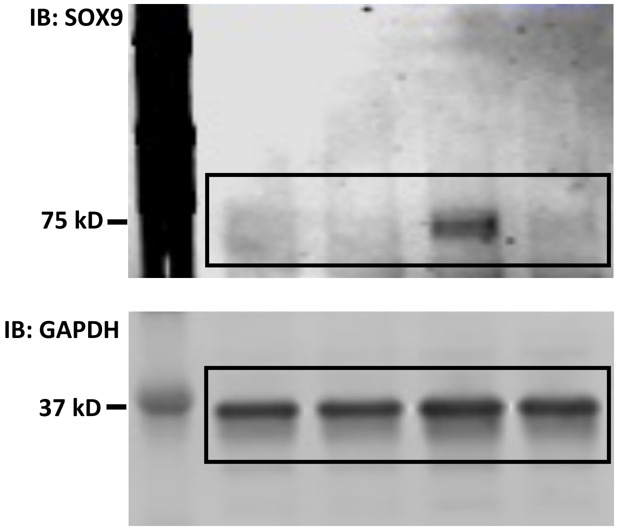

Fig6K

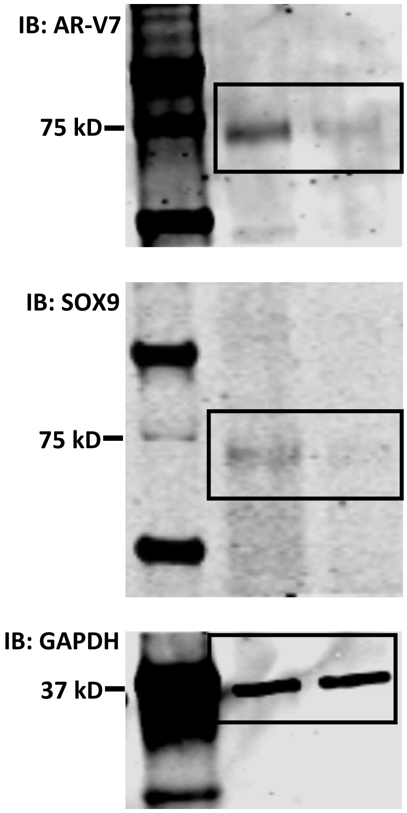

Fig6M

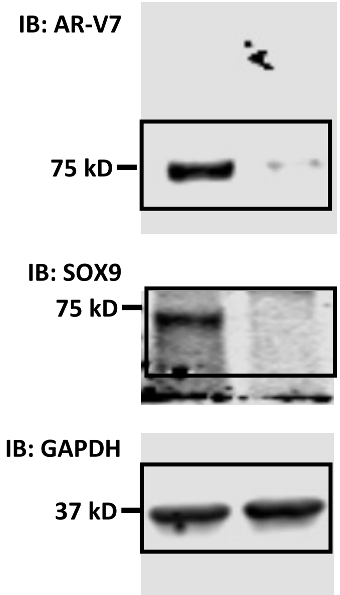

Fig7A

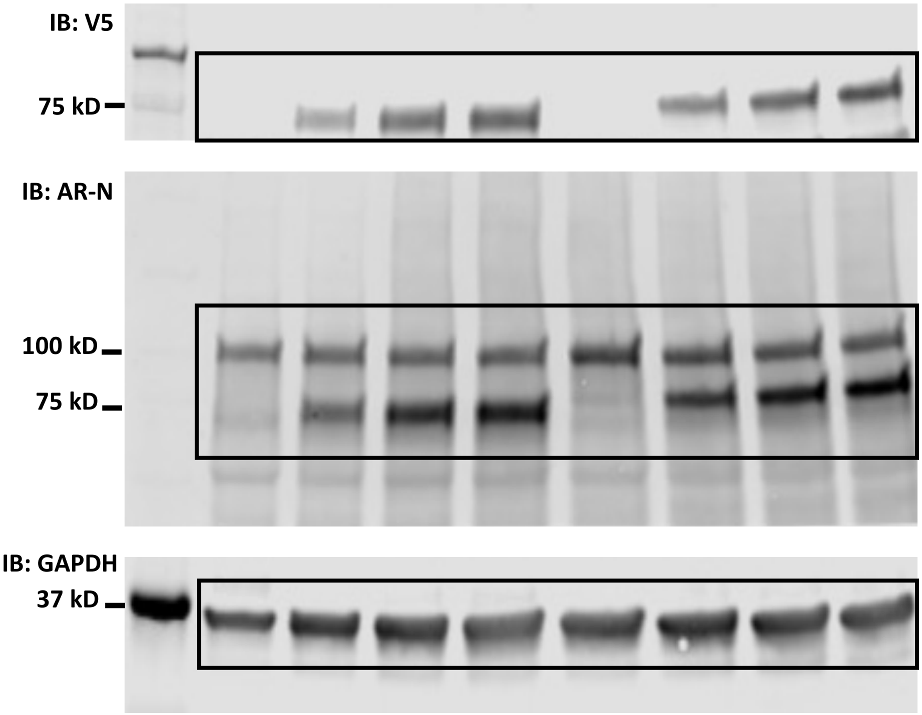

Fig8A

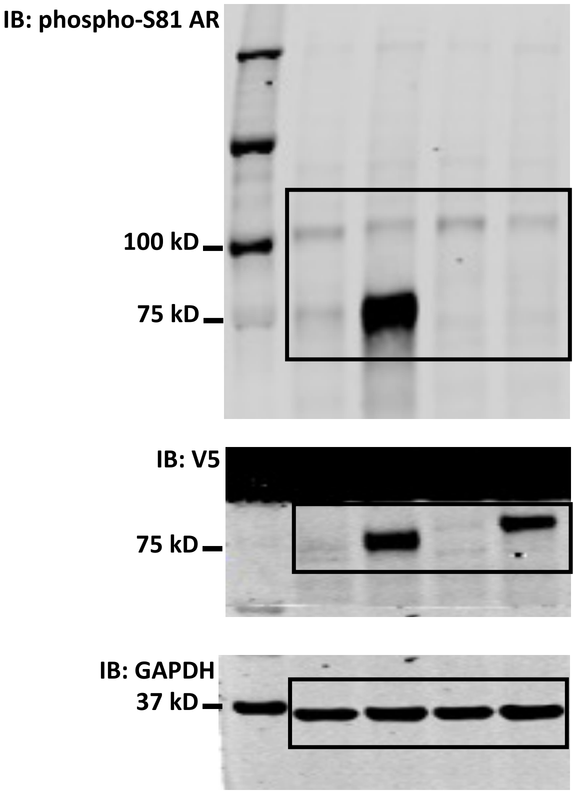

Fig8F

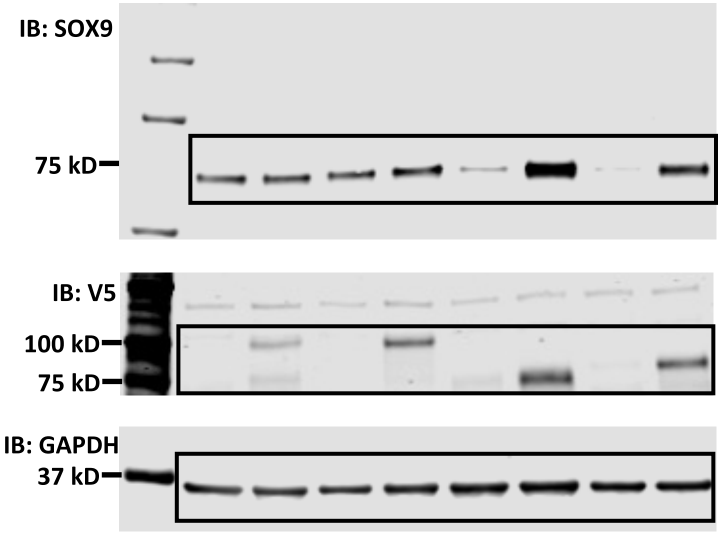

Fig9A

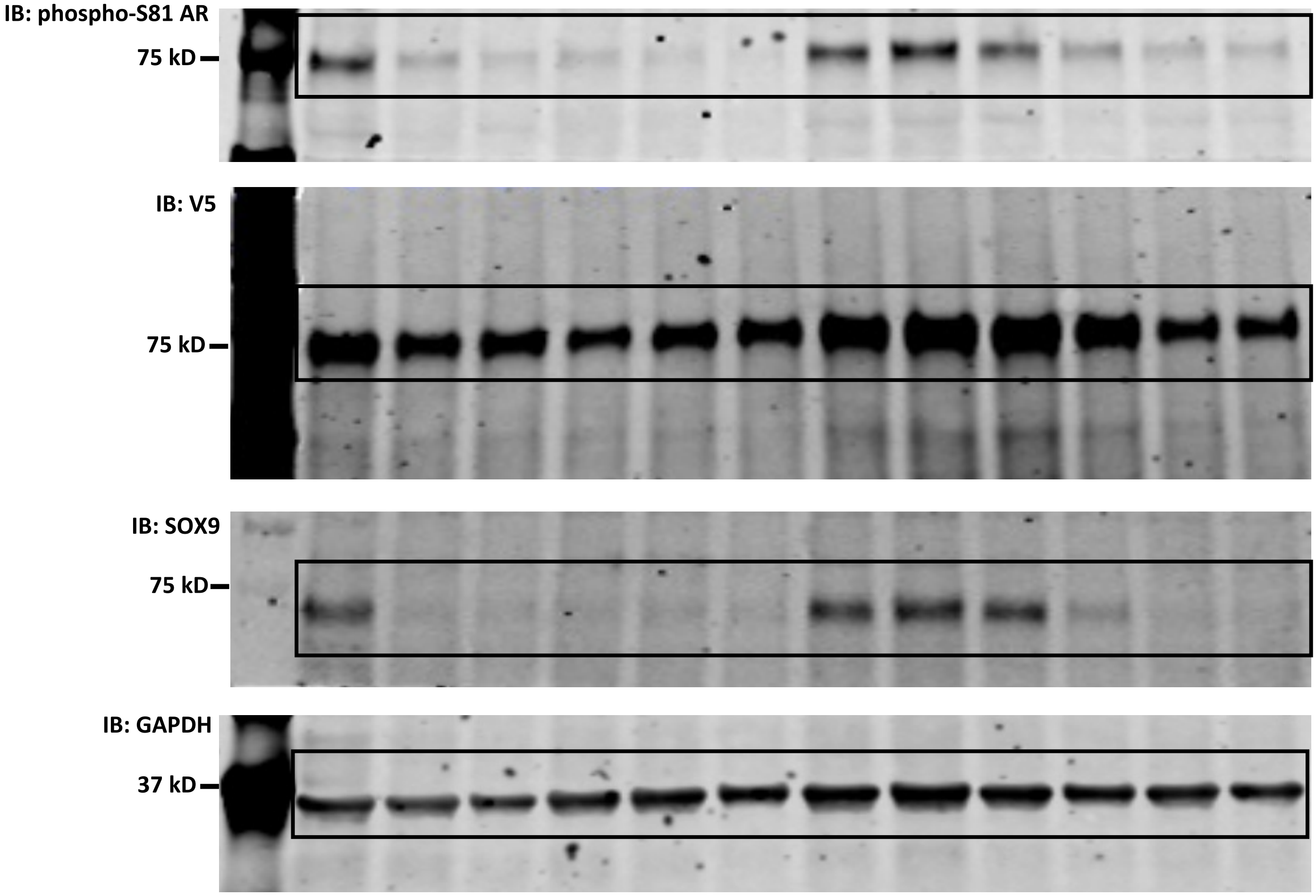

Fig9C

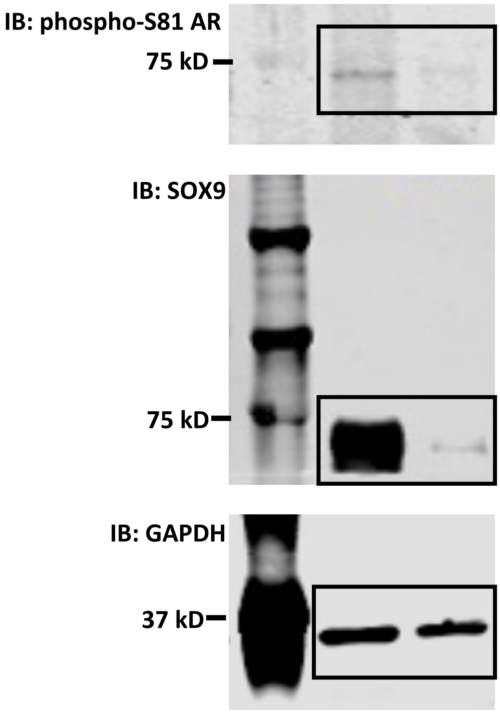

Fig9E

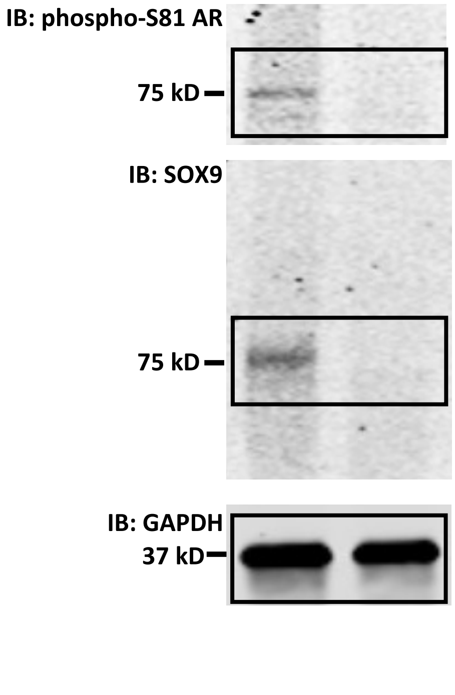

FigS1D

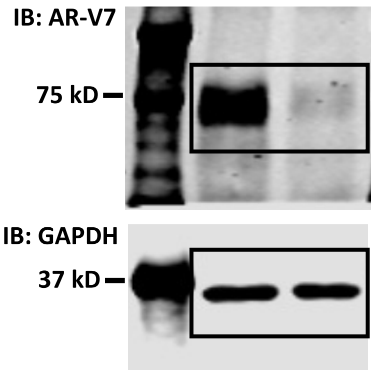

FigS2C

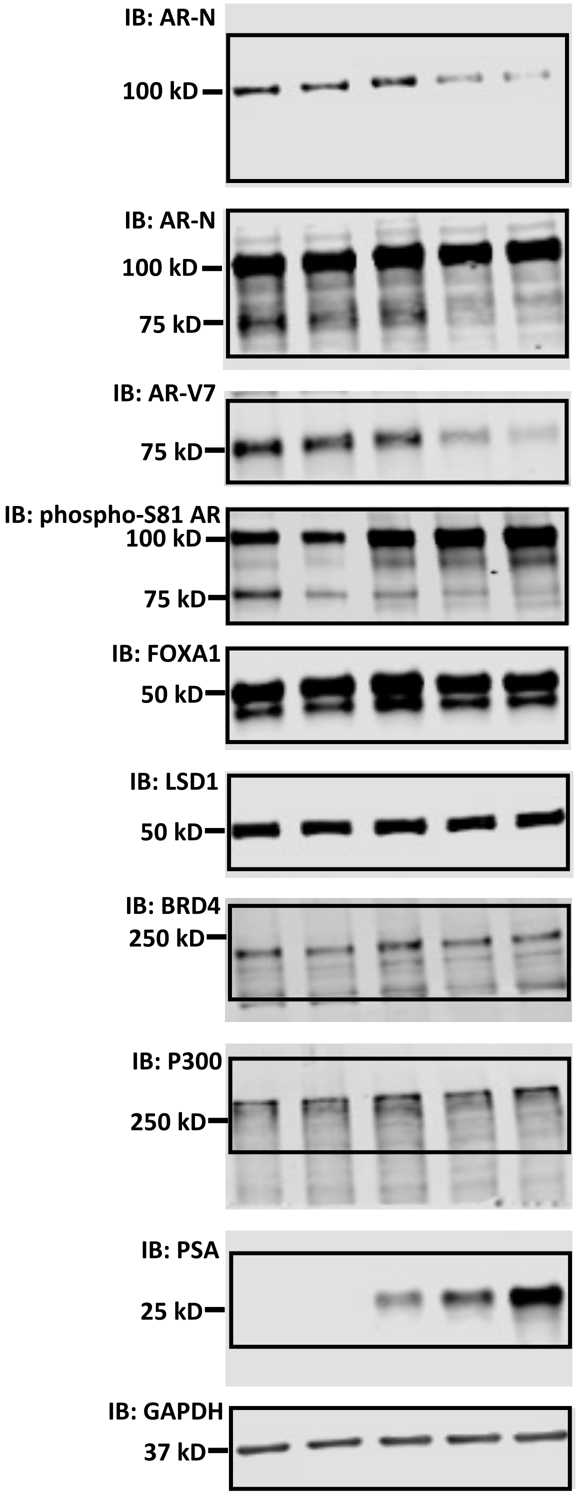

FigS8E

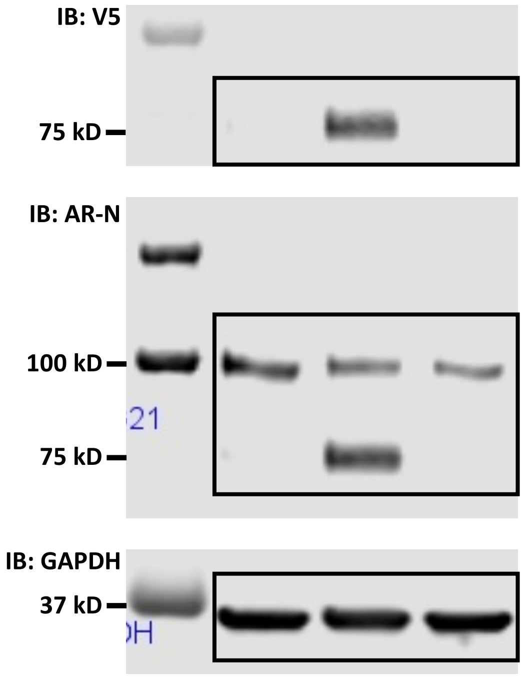

FigS9E

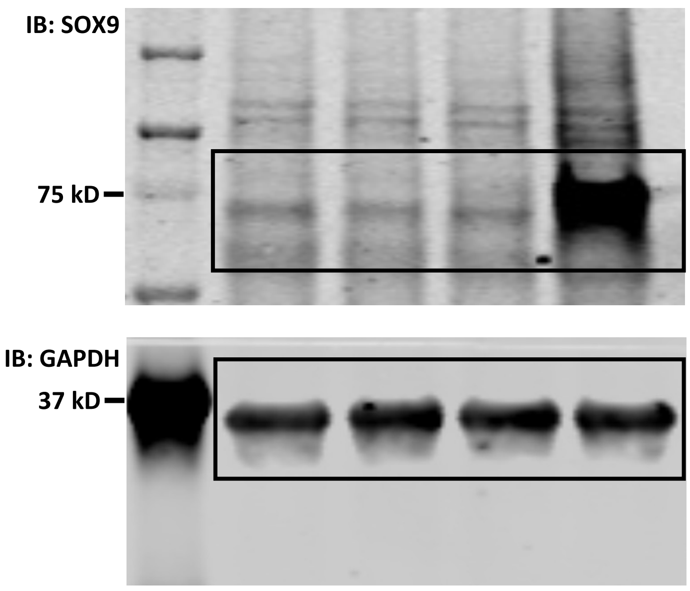

FigS10A

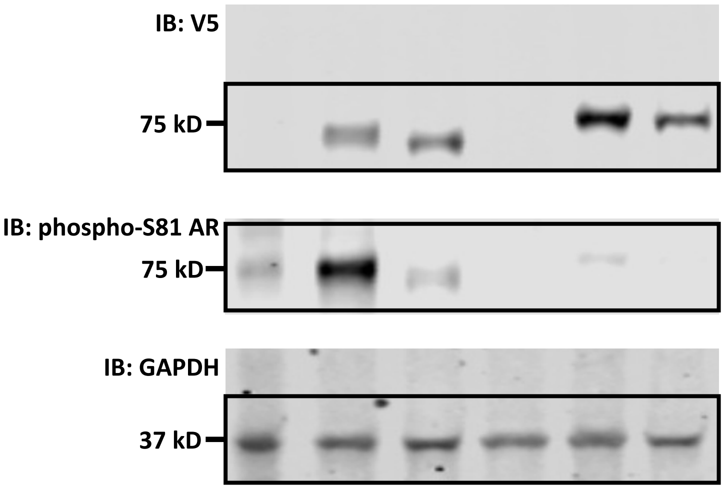

FigS10B

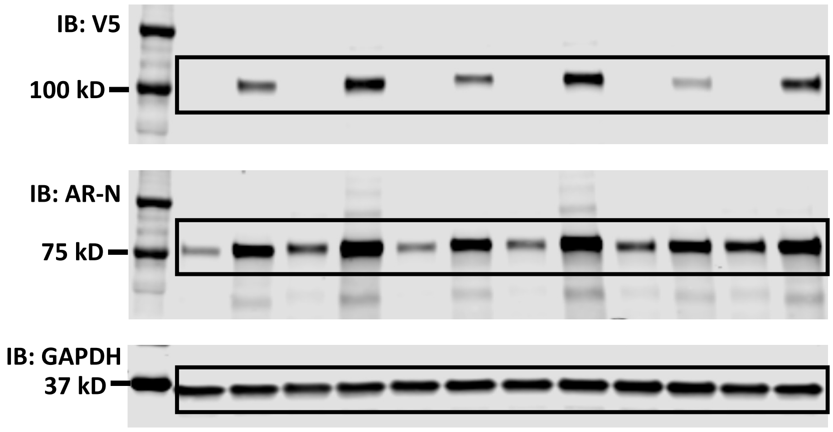

FigS11A

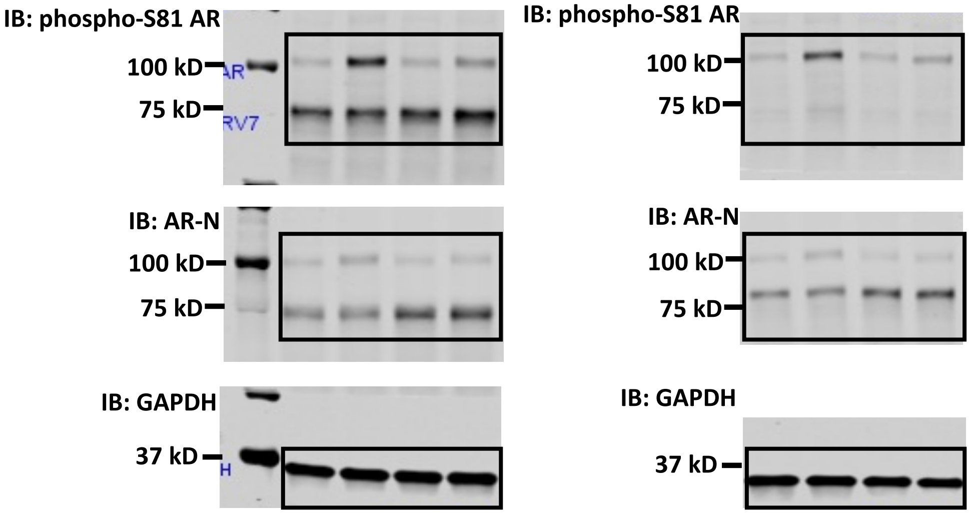

IB: phospho-S81 AR
100 kD
AR
75 kD
RV7
IB: AR-N
100 kD
75 kD
IB: GAPDH
37 kD
H
IB: phospho-S81 AR
100 kD
75 kD
IB: AR-N
100 kD
75 kD
IB: GAPDH
37 kD
